# SUMOylation substrate encoding genes as prognostic biomarkers in pancreatic ductal adenocarcinoma with functional assessment of *SAF-B2*

**DOI:** 10.3389/fphar.2025.1532658

**Published:** 2025-04-16

**Authors:** Xiangjun Wang, Chuanxin Yang, Yangming Liu, Jian Wang

**Affiliations:** Department of Hepatobiliary and Pancreatic Surgery, Shanghai Sixth People’s Hospital Affiliated to Shanghai Jiao Tong University School of Medicine, Shanghai, China

**Keywords:** Pancreatic ductal adenocarcinoma, SUMOylation, TAK-981, Prognosis, SAFB2, Wnt/β-Catenin signaling pathway

## Abstract

**Background:**

Pancreatic ductal adenocarcinoma (PDAC) is highly malignant with a poor prognosis, posing significant clinical challenges. SUMOylation, a reversible post-translational modification, plays a critical role in tumor progression, yet its prognostic significance in PDAC remains unclear.

**Methods:**

We assessed SUMOylation expression patterns and function in PDAC using Western blot and the SUMOylation inhibitor TAK-981. Differentially expressed SUMOylation substrate encoding genes (DE-SSEGs) were identified from The Cancer Genome Atlas (TCGA) and the Genotype-Tissue Expression Project (GTEx) datasets. A SUMOylation-based prognostic model, Sscore, was constructed using LASSO and Cox regression. Additional analyses included somatic mutation, immune infiltration, TIDE, drug sensitivity, and single-cell RNA sequencing. The role of *SAFB2* in PDAC was validated *in vitro*.

**Results:**

PDAC cells showed elevated SUMOylation, and its inhibition reduced cell proliferation. The Sscore model, based on DE-SSEGs (*CDK1*, *AHNAK2*, *SAFB2*), predicted overall survival and correlated with genome variation, immune infiltration, and drug sensitivity. Single-cell analysis further confirmed a link between high Sscore and malignancy. *SAFB2*, identified as a pivotal gene within the Sscore model, was significantly downregulated in PDAC tissues and cell lines; its overexpression was shown to inhibit PDAC cell proliferation, migration, and invasion by suppressing the Wnt/β-Catenin signaling pathway.

**Conclusion:**

This study underscores the role of SUMOylation in PDAC and introduces the Sscore as a prognostic tool. *SAFB2* is identified as a potential tumor suppressor, offering new therapeutic targets for PDAC.

## 1 Introduction

Pancreatic ductal adenocarcinoma (PDAC) is one of the most malignant solid tumors globally, with high mortality and poor prognosis, having a dismal 5-year survival rate of just 13%, and its incidence is rising annually (Conroy et al., 2023; Kumar et al., 2022; Siegel et al., 2023; Huang et al., 2021; Siegel et al., 2024). PDAC often remains asymptomatic in its early stages, progresses rapidly, and is frequently diagnosed in advanced stages, with 30%–35% of patients presenting with locally advanced disease and 50%–55% with metastasis (Conroy et al., 2023; Peng et al., 2019; Vincent et al., 2011). Surgical resection rates at diagnosis range from 10% to 20%, but the recurrence and metastasis rates post-surgery are as high as 80% (Siegel et al., 2024). As a result, neoadjuvant therapy such as chemotherapy plays a vital role in PDAC treatment, given that surgery alone does not significantly improve long-term survival (Siegel et al., 2024; Vincent et al., 2011). The latest National Comprehensive Cancer Network (NCCN) Guidelines Version 3.2024 recommend NALIRIFOX (Nanoliposomal Irinotecan, Leucovorin, Fluorouracil, and Oxaliplatin) as a first-line therapy for advanced PDAC patients (Conroy et al., 2023; Ma et al., 2024). Studies have shown that adjuvant systemic chemotherapy with gemcitabine can double the long-term survival rate (>5 years) for PDAC patients, with an associated 20% increase in cure rate (Sinn et al., 2017). However, advanced-stage PDAC remains a challenge, as the survival benefit from this treatment is limited. Furthermore, targeted therapies and immunotherapies that have proven effective in other malignancies show limited efficacy in PDAC. For instance, ipilimumab, a therapy approved for melanoma and renal cell carcinoma, has almost no effect on advanced PDAC, and CAR-T cell therapy, which has shown promise in lymphoma and leukemia, is also largely ineffective in PDAC (Li et al., 2018; Yoon et al., 2021). Therefore, improving early diagnosis rates and identifying novel therapeutic targets is crucial for better clinical outcomes in PDAC.

Small ubiquitin-related modifier (SUMOylation) is a type of protein translational modification mediated by SUMO proteins, which regulate the expression, localization, and activity of substrate proteins in numerous biological processes, including cell cycle regulation, metabolism, gene transcription, and DNA damage repair (Gu et al., 2023; Hendriks and Vertegaal, 2016). The SUMO protein family comprises SUMO1, SUMO2, and SUMO3, with SUMO2/3 showing up to 95% sequence and functional homology (Hendriks and Vertegaal, 2016). Similar to ubiquitination, SUMOylation is carried out through a three-step enzymatic cascade involving SUMO-activating enzyme E1 (SAE1/SAE2), SUMO-conjugating enzyme E2 (UBC9), and SUMO-ligating enzyme E3 (Gu et al., 2023; Hendriks and Vertegaal, 2016; Vertegaal, 2022; Han et al., 2018). Additionally, deSUMOylation enzymes, such as those from the SENP family (SENP1, 2, 3, 5, 6, and 7), play a crucial role in regulating SUMOylation levels (Wu and Huang, 2023). Recent studies have shown that SUMOylation is upregulated in several solid tumors, including breast, colorectal, prostate, lung, and pancreatic cancers, and its overexpression correlates with poor patient prognosis (Seeler and Dejean, 2017; Li et al., 2022). Given the diversity of SUMO-modified substrate proteins, SUMOylation can regulate tumorigenesis and progression by influencing cell cycle regulation, DNA damage response, genomic instability, tumor metabolism, and immune evasion (Vertegaal, 2022; Sun et al., 2023). Understanding the role of SUMOylation in PDAC and investigating its potential as a target for therapy is therefore critical.

Scaffold attachment factor-B2 (*SAFB2*) is a nuclear matrix-associated protein belonging to the *SAFB* family, which is widely expressed across human tissues (Renz and Fackelmayer, 1996; Hong et al., 2012). The family includes three members: *SAFB1*, *SAFB2*, and SAF-like transcription modulator (*SLTM*) (Hong et al., 2012). *SAFB2* plays roles in processes such as RNA post-transcriptional processing, cell proliferation, stress response, and apoptosis (Hutter et al., 2020). In hormone-dependent cancers, including breast and prostate cancers, *SAFB2* expression is downregulated and delays tumor progression by inhibiting the transcriptional activities of androgen receptors (AR) and estrogen receptors α (ERα) (Zhen et al., 2023). While the expression and function of *SAFB2* in PDAC remain understudied, its established tumor-suppressive role suggests that it may be critical in PDAC progression. Further exploration of the specific function of *SAFB2* in PDAC could enhance our understanding of its biology and offer novel therapeutic strategies.

In this study, we found that SUMOylation levels were significantly elevated in PDAC cells and tissues, and inhibiting SUMOylation reduced PDAC cell proliferation, confirming the critical role of SUMOylation in PDAC progression. Using SUMOylation proteomics data compiled by Ivo A et al., we integrated transcriptomic data from tumor and normal tissues in The Cancer Genome Atlas (TCGA) and the Genotype-Tissue Expression Project (GTEx) to develop a SUMOylation substrate encoding gene prognostic model for PDAC (Hendriks and Vertegaal, 2016). This model showed robust predictive accuracy in assessing patient prognosis, immune cell infiltration in the tumor microenvironment, genomic variation, and drug sensitivity. Additionally, *SAFB2*, a key SUMOylation substrate encoding gene in this model, was significantly downregulated in PDAC tissues and cell lines. Further *in vitro* studies revealed that overexpression of *SAFB2* significantly inhibited the proliferation, invasion, and migration of PDAC cells by suppressing the Wnt/β-Catenin signaling pathway, indicating its potential role as a tumor suppressor in PDAC. Overall, our study establishes a novel three-gene SUMOylation substrate prognostic model and highlights the important tumor-suppressive function of *SAFB2* in PDAC.

## 2 Materials and methods

### 2.1 Patients and specimens

This study included samples from patients diagnosed with primary PDAC at Shanghai Sixth People’s Hospital between 6 January 2025, and 14 March 2025. A total of five patients were enrolled, and matched adjacent normal tissues were collected for comparative analysis. Informed consent was obtained from all participants, and the study was approved by the Ethics Committee of Shanghai Sixth People’s Hospital.

### 2.2 Data extraction and patient information preprocessing

Transcriptome data and corresponding clinical features were obtained from the TCGA-PAAD cohort (n = 181) within the TCGA database (https://portal.gdc.cancer.gov) for analysis. Transcriptomic data from normal pancreatic tissue (n = 168) in the GTEx database (https://www.gtexportal.org/) served as controls. To minimize batch effects, the “limma” package was employed for normalization. To further validate the stability of the results, gene expression matrix files, along with clinical and survival data, were downloaded the GSE62452 (doi: 10.1158/0008-5472; n = 65), GSE183795 (doi: 10.1093/carcin/bgac092; n = 134) datasets from the GEO database (https://www.ncbi.nlm.nih.gov/geo/) (Yang et al., 2016; Yang S. et al., 2022).

### 2.3 Selection and annotation of SUMOylation substrate encoding genes

Based on the SUMO target protein list provided by Hendriks and Vertegaal, (2016), 334 proteins with mass spectrometry scores ≥15 were selected as SUMOylation substrate encoding genes (SSEGs) (Hendriks and Vertegaal, 2016). Differential gene expression analysis between PDAC samples from the TCGA database and normal control samples from the GTEx database was conducted using the “limma” package, identifying 5,871 differentially expressed genes (DEGs) with |log2 Fold change (FC)| > 1 and adjusted p value <0.05. By intersecting the SSEGs and DEGs lists, 134 differentially expressed SUMOylation substrate encoding genes (DE-SSEGs) between normal and tumor samples were identified. Volcano plots and heatmaps were generated based on FC and adjusted p values to visualize these DEGs. All DE-SSEGs were then uploaded to the Search Tool for Retrieval of Interacting Genes database (STRING, https://string-db.org/) for protein-protein interaction (PPI) analysis, and Cytoscape software was used for network visualization. The CytoHubba plugin was applied to rank the DE-SSEGs based on their PPI, with the top 30 hub genes being selected. Functional annotation analysis, including Gene Ontology (GO) and Kyoto Encyclopedia of Genes and Genomes (KEGG) pathway enrichment, was conducted using the “ClusterProfiler” package to determine the potential functions of these genes, with the significance threshold set at p < 0.05.

### 2.4 Immunohistochemistry of prognostic hub genes

We verified the protein expression of key prognostic genes in tumor and normal tissues using Immunohistochemistry (IHC) data from the Human Protein Atlas (HPA) database (https://www.proteinatlas.org/).

### 2.5 Construction and validation of prognostic biomarkers based on SUMOylation substrate encoding genes

Univariate Cox regression analysis was performed to identify DE-SSEGs significantly associated with overall survival (OS). Subsequently, a prognostic model was developed in the training cohort based on DE-SSEGs using the least absolute shrinkage and selection operator (LASSO) Cox regression and stepwise multivariate Cox proportional hazards regression analysis. Patients were categorized into High- and Low-Sscore groups based on the median score. The performance of model was evaluated through receiver operating characteristic (ROC) curves, Kaplan-Meier (K-M) survival curves, and risk score distribution plots.

### 2.6 Calibration curve and nomogram construction

The “Root Mean Square (RMS)” package was utilized to construct a nomogram integrating risk scores and other clinicopathological features to estimate the individual survival probability. The cumulative score was used to estimate each patient’s survival prognosis (Xiang et al., 2020). Calibration curves were plotted to assess the consistency between predicted and observed survival outcomes, and ROC analysis was used to evaluate the predictive performance of the nomogram.

### 2.7 Immune infiltration analysis and immunotherapy response prediction

The Estimation of STromal and Immune cells in MAlignant Tumours using Expression data (ESTIMATE) algorithm was used to calculate immune scores, stromal scores, ESTIMATE scores, and tumor purity for the samples (Xiang et al., 2020; Yoshihara et al., 2013). Additionally, the relative abundance of various immune cell types was estimated using the cell-type identification by estimating relative subsets of RNA transcripts (CIBERSORT) and single sample gene set enrichment analysis (ssGSEA) methods, implemented through the “CIBERSORT” and “Gene Set Variation Analysis (GSVA)” packages, respectively. The tumor immune dysfunction and exclusion (TIDE) algorithm was applied to predict the potential response of PDAC patients to immune checkpoint inhibitors (ICIs) (Jiang et al., 2018).

### 2.8 Somatic mutation and copy number variation analysis

Somatic mutation data were obtained from the TCGA database, with the tumor mutation burden (TMB) defined as the total number of somatic coding errors, base substitutions, and indel mutations per megabase. Commonly mutated genes in PDAC samples were visualized using the “Maftools” package, and waterfall plots were generated to illustrate the mutation landscape of individual patients (Mayakonda et al., 2018). The GRCh38 reference genome was used for annotating genes in copy number variation (CNV) regions.

### 2.9 Prediction of chemotherapeutic drug sensitivity

Drug sensitivity for each sample was predicted using the “OncoPredict” package, with the half-maximal inhibitory concentration (IC50) values calculated for common targeted and chemotherapeutic drugs (Maeser et al., 2021). IC50 data were sourced from the Cancer Therapeutics Response Portal (CTRP, https://portals.broadinstitute.org/ctrp/).

### 2.10 Single-cell RNA sequencing (scRNA-seq) quality control and analysis

We analyzed normalized scRNA-seq data from six PDAC tissues and three adjacent noncancerous pancreatic tissues (GSE212966,GSE194247) (Satija et al., 2015; Chen et al., 2023). scRNA-seq data were processed using the “Seurat” (v5.0.1) package, with low-quality cells (gene count per cell <500, mitochondrial gene percentage >15%, and cell count per gene 500 < n < 5000) filtered out (Hao et al., 2021). Normalization was performed using the “NormalizeData” function (LogNormalize). Two thousand highly variable genes were selected for principal component analysis (PCA) to achieve dimensionality reduction, followed by uniform manifold approximation and projection (UMAP) for visualization. Doublets were identified using the “DoubletFinder” (v2.0.3) package, assuming a doublet rate of 5% for the droplet channel of each sample (McGinnis et al., 2019). Additionally, gene integration from different samples was performed using the “Harmony” package (Korsunsky et al., 2019). Cell clusters were identified by matching cluster-specific genes with known cell type markers reported in the literature and the CellMarker database (http://xteam.xbio.top/CellMarker/) (Hao et al., 2021).

### 2.11 Single-cell CNV inference

The “inferCNV” (v1.6.0) package was used to infer large-scale somatic CNVs (Patel et al., 2014). In brief, the gene expression matrix, annotation data, and gene/chromosome location information of ductal cells were analyzed. All other cells were treated as reference cells without CNVs. CNV scores for each cell cluster were computed by calculating the second-order sum of CNV regions.

### 2.12 Construction of single-cell trajectories in PDAC

Single-cell trajectory analysis was performed using the “Monocle2” (v2.28.0) package to reveal the dynamic changes in cell states (Trapnell et al., 2014). The abnormal gene expression profile of ductal cells was set as the root_state parameter to execute cell ordering, determining the pseudotime starting point. Dimensionality reduction was carried out using the “DDRTree” algorithm, and a minimum spanning tree was visualized using the “plot_cell_trajectory” function.

### 2.13 GSVA analysis in scRNA-seq

GSVA was conducted using the “ClusterProfiler” package to compare pathway activation differences between groups (Wu et al., 2021). All pathway data were obtained from the Molecular Signatures Database (MSigDB).

### 2.14 Cell lines

Human PDAC cell lines, including CAPAN-1, CFPAC1, MIA-PaCa2, PANC-1, and AsPC-1, as well as normal pancreatic duct epithelial cell line HPNE and human embryonic kidney cell line HEK293T, were obtained from the American Type Culture Collection (ATCC, Manassas, VA). Cells were cultured in media supplemented with 10% fetal bovine serum and 1% penicillin/streptomycin. HPNE, MIA-PaCa2, and PANC-1 cells were cultured in DMEM/F12 (GIBCO); BxPC-3 and AsPC-1 cells in RPMI 1640 (GIBCO); and CAPAN-1 and CFPAC1 cells in IMDM (GIBCO). All cell lines were routinely tested for *mycoplasma* contamination.

### 2.15 RNA extraction and gene/protein expression analysis

Total RNA from pancreatic cells and tissues was extracted using Trizol reagent (Invitrogen™) according to the manufacturer’s instructions. ABScript III RT Master Mix (ABclonal, RK20428) and Universal SYBR Green Fast qPCR Mix (ABclonal, RK21203) were used for subsequent analyses. The primer sequences for 18S were: forward, 5′-TTC​GAA​CGT​CTG​CCC​TAT​CAA-3′; reverse, 5′-ATG​GTA​GGC​ACG​GCG​ACT​A-3′. The primer sequences for CDK1 were: forward, 5′-TAC​AGG​TCA​AGT​GGT​AGC​CAT​GAA-3′; reverse, 5′-GCA​TAA​GCA​CAT​CCT​GAA​GAC​TGA-3′. The primer sequences for AHNAK2 were: forward, 5′-TTC​AGA​GCC​GTA​CAA​GGT​TCA​GT-3′; reverse, 5′-CAG​CAA​CAT​CCG​TGT​CCT​CCT-3′. The primer sequences for AHNAK2 were: forward, 5′- GAA​GCC​ACC​AGC​AAG​AAG​TCA​G-3′; reverse, 5′-TCG​TCT​AGC​ACA​CTC​ATG​TCC​AT-3′.

Protein expression levels were assessed by Western blot analysis. Proteins from collected cell samples were first separated by 10% SDS-polyacrylamide gel electrophoresis (SDS-PAGE) and subsequently transferred to a polyvinylidene fluoride (PVDF) membrane (IPVH00010, Sigma). To block nonspecific binding, the membrane was incubated at room temperature for 2 h in 5% non-fat milk. Afterward, the membrane was incubated with the primary antibody overnight. Following washing, the membrane was further incubated for 1 h with horseradish peroxidase (HRP) - conjugated goat anti-rabbit and anti-mouse IgG secondary antibodies (1:25,000, E030110-01, E030120-01, EARTHOX). Finally, protein signals were detected and visualized using chemiluminescence. Antibody against SUMO1 (SinoBiological, 13095-RP02, dilution 1:1,000), SUMO2/3 (Cell Signaling Technology, 4971S, dilution 1:1,000), and SAFB2 (Proteintech, 11642-1-AP, dilution 1:1,000), β-catenin (Abmart, M24002F, dilution 1:1,000), c-MYC (Abamart, T55150F, dilution 1:1,000), and CCND1 (MCE, HY-P80633, dilution 1:1,000).

### 2.16 Lentivirus production and transduction

For the construction of stable *SAFB2*-overexpressing cell lines, lentiviral vectors carrying the SAFB2 gene sequence in the pLV3-CMV-3×FLAG-SAFB2(human)-Puro construct were co-transfected with packaging plasmids (psPAX2 and pMD2.G) into 293T cells. The viral supernatants were collected, concentrated, and used to infect PDAC cell lines Capan-1 and AsPC-1. Infected cells were selected using puromycin for 1 week to generate stable cell lines.

### 2.17 Cell function assays

For the proliferation assay, cells (2,000 per well) were seeded into a 96-well plate containing 100 μL of complete medium and incubated at 37°C. At 0, 24, 48, 72, and 96 h, 10 μL of CCK-8 (C0038, Beyotime) reagent was added to each well. After a 2-h incubation, absorbance was measured at 450 nm. For the colony formation assay, cells were cultured in complete medium for 14 days, fixed with 4% paraformaldehyde for 15 min, and stained with 0.1% crystal violet for 10 min. In the drug sensitivity assay, cells were seeded under the same conditions into a 96-well plate (2,000 cells per well) and continuously treated with TAK-981 (paraformaldehyde) at specified concentrations, with 0.1% DMSO used as the control (Kumar et al., 2022). Proliferation and colony formation assays were then conducted. In the Transwell migration assay, 5 × 10^4 cells were seeded in the upper chamber of a Transwell system and incubated for 24 h. For the invasion assay, cells were suspended in serum-free medium and seeded into Matrigel-coated Transwell chambers. After 48 h, cells that had migrated to the lower chamber were fixed with 4% paraformaldehyde and stained with 0.1% crystal violet for 10 min.

### 2.18 Cell cycle analysis

Cell cycle analysis was conducted using the Cell Cycle and Apoptosis Analysis Kit (C1052, Beyotime, China) following the manufacturer’s protocol. Red fluorescence signals were measured by flow cytometry with an excitation wavelength of 488 nm, and at least 10,000 events were collected per sample.

### 2.19 Statistical analysis

Statistical analyses were conducted using R software (version 4.3.1) and GraphPad Prism software (version 9). Survival analysis used the Kaplan–Meier method and Cox proportional hazards model. For normally distributed data, Student’s t-test and one-way ANOVA were applied for two-group and multiple-group comparisons, respectively. Non-normally distributed data were analyzed with the Mann–Whitney U test for two groups, and the Kruskal–Wallis for multiple groups. Categorical variables were assessed using the chi-squared or Fisher’s exact test as appropriate. All p-values were two-sided, with p < 0.05 considered statistically significant.

## 3 Results

### 3.1 Elevated SUMOylation level in tumor and reduction of proliferation by SUMOylation inhibition

**FIGURE 1:**
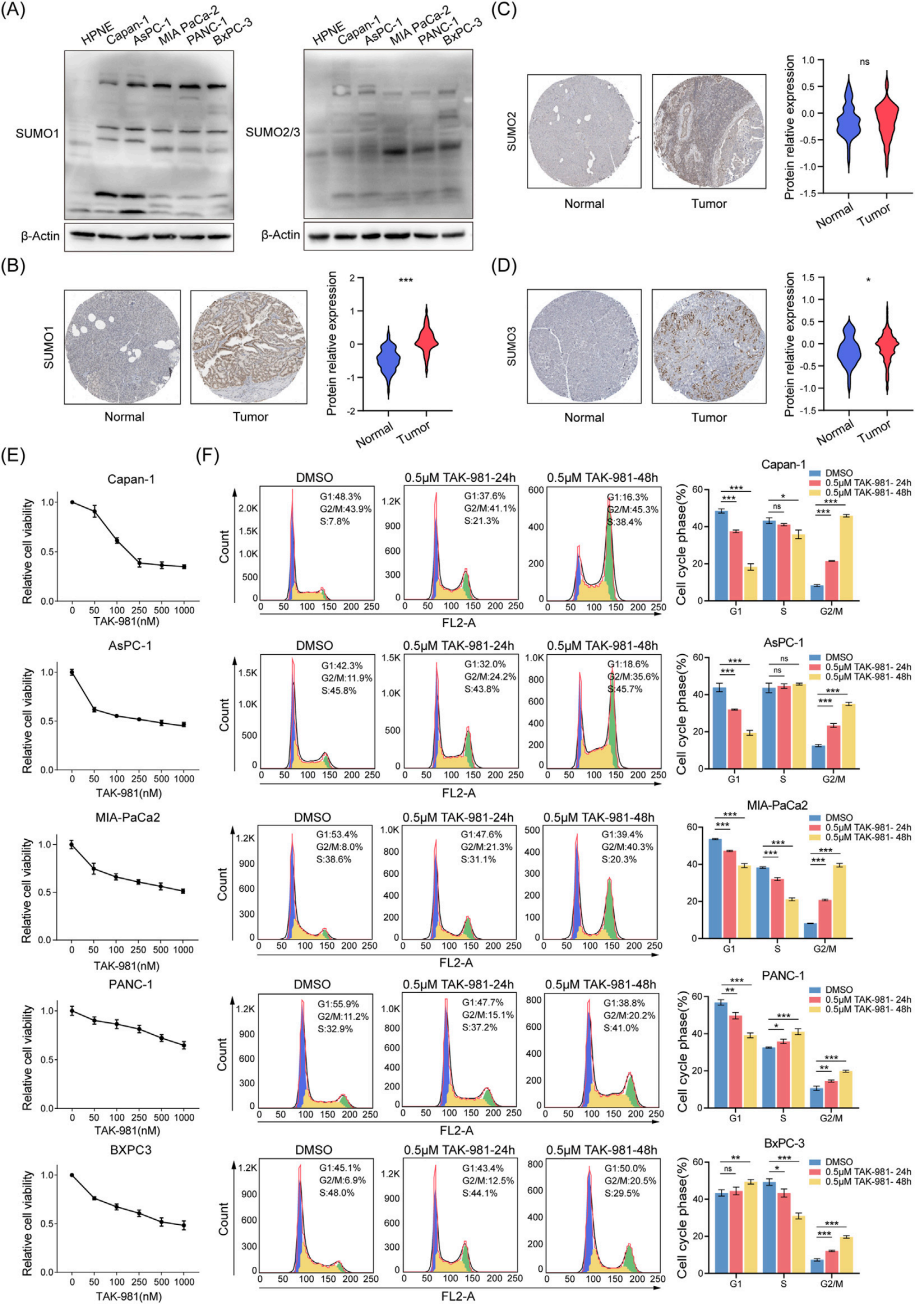
Elevated SUMOylation in PDAC and its inhibition impairs proliferation. **(A)** Western blot analysis shows SUMO1、SUMO2/3 protein levels in PDAC cell lines (Capan-1, AsPC-1, MIA-PaCa2, PANC-1, BxPC-3) and normal pancreatic duct epithelial cells. **(B–D)** Representative IHC images display SUMO1 **(B)**, SUMO2 **(C)** and SUMO3 **(D)** expression in normal pancreatic tissue and PDAC tissue from the HPA dataset. The violin plots represent relative quantification of SUMO protein expression in tissues, p-values were derived from Student’s t-tests. **(E)** Proliferation curves of PDAC cell lines treated with graded concentrations of TAK-981, error bars represent standard deviation (SD) based on three independent experiments. **(F)** PDAC cells lines (Capan-1, AsPC-1, MIA-PaCa2, PANC-1, BxPC-3) were treated with either 0.5 μM TAK-981 or 0.05% DMSO for 24 and 48 h, followed by cell cycle analysis. The bar graph represents the percentage of cells in each phase of the cell cycle (G1, S, and G2/M) based on three independent biological replicates (n = 3). Error bars represent SD, and p-values were derived from Student’s t-tests. ^ns^p > 0.05, *p < 0.05, **p < 0.01, ***p < 0.001.

We investigated the overall levels and potential functions of SUMOylation in PDAC. Western blot analysis showed that, compared to normal pancreatic ductal epithelial cells (hTERT-HPNE), the levels of pan-SUMOylation (SUMO1 and SUMO2/3) were markedly elevated in PDAC cell lines (Capan-1, AsPC-1, MIA-PaCa2, PANC-1, BxPC-3) (Figure 1A). IHC data from HPA further confirmed that pan-SUMOylation levels in PDAC tissues were significantly higher than those in normal pancreatic tissues (Figures 1B–D). To explore the effects of reducing SUMOylation, we treated PDAC cell lines with the small-molecule SUMOylation inhibitor TAK-981 (Kumar et al., 2022). As TAK-981 concentration increased, the proliferation of PDAC cells was significantly inhibited (Figure 1E). Furthermore, PDAC cell lines all showed a significant increase in the proportion of cells in the G2/M phase at both 24 and 48 h, indicating G2/M phase arrest (Figure 1F). These findings indicate that SUMOylation levels are highly elevated in PDAC cells, and inhibition via TAK-981 can effectively suppress their proliferation *in vitro*.

### 3.2 Development and evaluation of prognostic models based on DE-SSEGs

**FIGURE 2:**
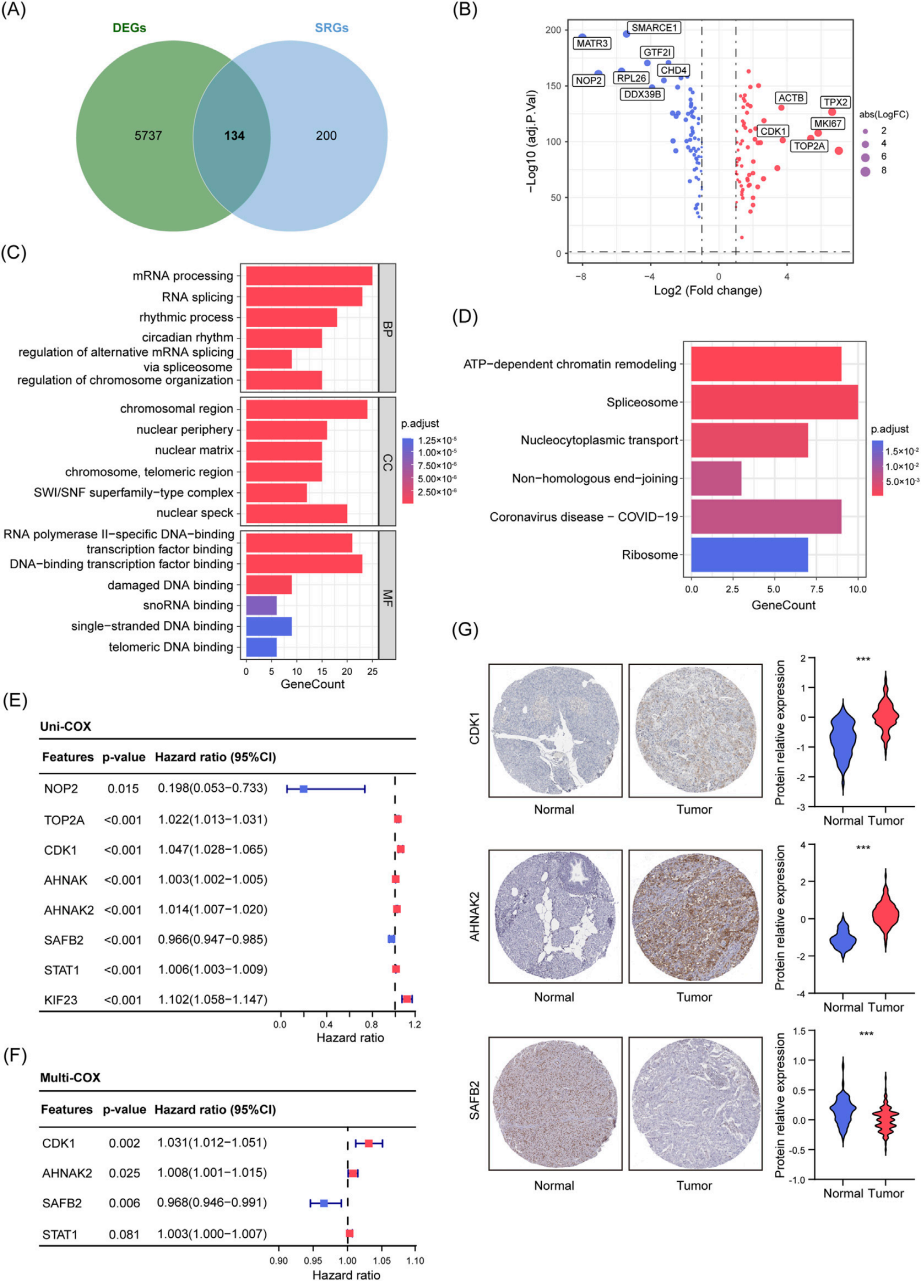
Screening, Functional Enrichment, and Prognostic Analysis of DE-SSEGs in PDAC. **(A)** Venn diagram showing the overlap between DEGs from tumor and normal tissues (n = 5871) and the SSEGs set (n = 334). **(B)** Volcano plot illustrating 134 DE-SSEGs based on adjusted p-values and FC. Red dots indicate upregulated genes, while blue dots indicate downregulated genes (|log2(FC)| >1, p < 0.05). **(C, D)** GO enrichment analysis **(C)** and KEGG pathway enrichment analysis **(D)** of DE-SSEGs. **(E, F)** Forest plots from univariate **(E)** and multivariate **(F)** Cox regression analyses of DE-SSEGs. **(G)** Representative IHC images showing *CDK1*, *AHNAK2*, and *SAFB2* expression in normal pancreatic and PDAC tissues. The violin plots represent relative quantification of protein relative expression in tissues, p-values were derived from Student’s t-tests. ***p < 0.001.

To further explore the potential roles of SUMOylation in PDAC, we used SSEGs as prognostic markers for stratifying PDAC patients. First, we performed differential expression analysis using transcriptomic data from PDAC tissues and normal pancreatic tissues/adjacent normal tissues from the TCGA and GTEx databases, identifying 5,871 DEGs. These DEGs were then intersected with highly enriched SUMOylation proteomics data compiled by Ivo A. et al. (enrichment score ≥15), ultimately identifying 134 DE-SSEGs in PDAC and normal tissues (Figures 2A, B; Supplementary Figure S1A; Hendriks and Vertegaal, 2016). We further used the STRING interaction gene database to conduct PPI network analysis on DE-SSEGs, selecting the top 30 key genes for analysis. The PPI analysis suggested that key genes such as *TP53BP1*, *HNRNPA1*, *PARP1*, and *NPM1* are central to processes like DNA damage repair, cell cycle regulation, and post-transcriptional modification (Supplementary Figure S1B). Additionally, GO and KEGG enrichment analyses revealed that these genes are significantly enriched in processes related to chromatin remodeling, RNA splicing, and nucleocytoplasmic transport, highlighting their critical role in gene expression regulation, maintenance of genomic stability, and cellular stress response (Figures 2C, D).

After identifying the functional significance of DE-SSEGs in PDAC, we developed a prognostic model to pinpoint key genes closely related to patient survival, offering insights valuable for clinical decision-making. Initially, we performed univariate Cox regression and LASSO regression on 134 DE-SSEGs, identifying eight genes significantly correlated with OS (Supplementary Figures S1C, D; Figure 2E). To enhance the interpretability of model, multivariate Cox regression was conducted, narrowing down three key prognostic genes: *CDK1*, *AHNAK2*, and *SAFB2* (p < 0.05) (Figure 2F). Next, using data from the HPA, we validated the expression levels of CDK1, AHNAK2, and SAFB2 in PDAC and normal tissues. IHC results demonstrated significant upregulation of CDK1 and AHNAK2 in PDAC tissues, while SAFB2 was notably downregulated (Figure 2G), confirming their relevance in PDAC prognosis. Based on these genes, we constructed a risk score model (Sscore).

$$Sscore=1.3987\;*\;\exp\left[\left(-0.0340\right)\;*\;SAFB2+\left(0.0338\right)\;*\;CDK1+\left(0.0085\right)\;*\;AHNAK2\right]$$

**FIGURE 3:**
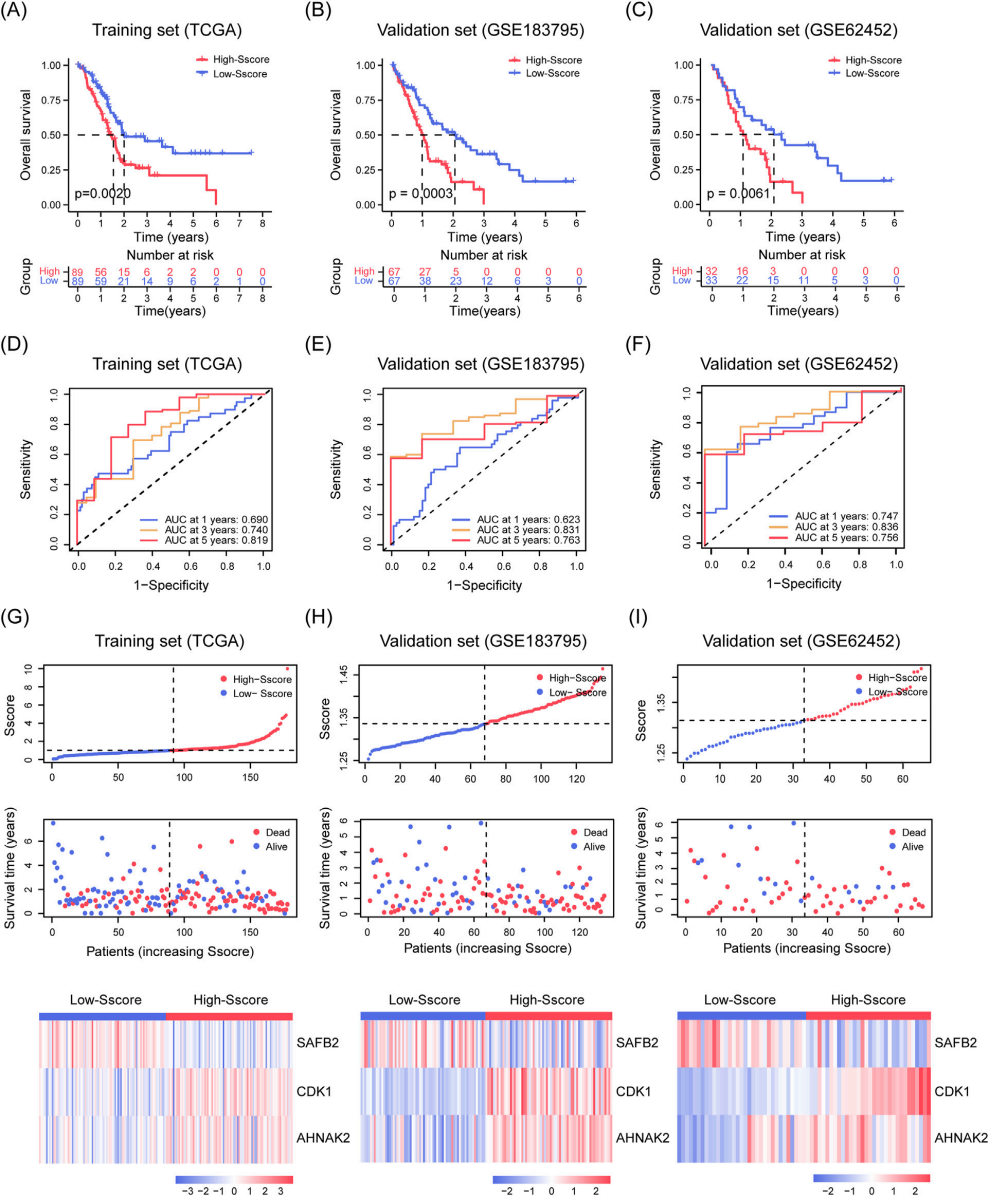
Stability and Predictive Performance of the Sscore Model. **(A–C)** K-M analysis illustrating OS curves for high- and low-Sscore patients in the TCGA-PAAD training set **(A)** and in the GSE183795 **(B)** and GSE62452 **(C)** validation sets. **(D–F)** ROC curves assessing the OS prediction in the TCGA-PAAD training set **(D)** and in GSE183795 **(E)** and GSE62452 **(F)** validation sets for SUMOylation substrate encoding gene features. **(G–I)** Scatter plots showing risk scores (top), survival time and status (middle), and heatmap of CDK1, AHNAK2 and SAFB2 expression (bottom) for PDAC patients in the TCGA-PAAD training set **(G)**, GSE183795 **(H)**, and GSE62452 **(I)** validation sets.

We first calculated the risk scores for PDAC patients from the training cohort (TCGA-PAAD, n = 178) and two validation cohorts (GSE183795, n = 134; GSE62452, n = 65) (Yang et al., 2016; Yang S. et al., 2022). Using the median score as a cutoff, patients were categorized into High- and Low-Sscore groups. K-M analysis showed that patients in the Low-Sscore group had significantly better OS in the training cohort (p = 0.0020, Figure 3A) and both validation cohorts (p = 0.0003 and p = 0.0061, Figures 3B, C). Similarly, time-dependent ROC analyses for 1-, 3-, and 5-year OS in the training (Figure 3D) and validation cohorts (Figures 3E, F) reinforced the prognostic power of the Sscore model in PDAC. Figures 3G–I depict the relationships between Sscore, survival times, and patient status, along with the transcriptional profiles of *CDK1*, *AHNAK2*, and *SAFB2* across the High- and Low-Sscore groups.

To evaluate the prognostic efficacy of the Sscore in other gastrointestinal malignancies, we first observed that global SUMOylation levels were significantly elevated in LIHC and COAD compared to normal tissues (Supplementary Figures S2A, B). K-M survival analysis and ROC curve assessment demonstrated that the Sscore was only effective in predicting overall survival in LIHC patients (P = 0.002), although its prognostic performance was substantially inferior to that observed in PDAC (Supplementary Figures S2C–F). These findings further establish the significance of the Sscore in PDAC prognosis.

### 3.3 Construction and validation of a prognostic nomogram based on sscore for PDAC

**FIGURE 4:**
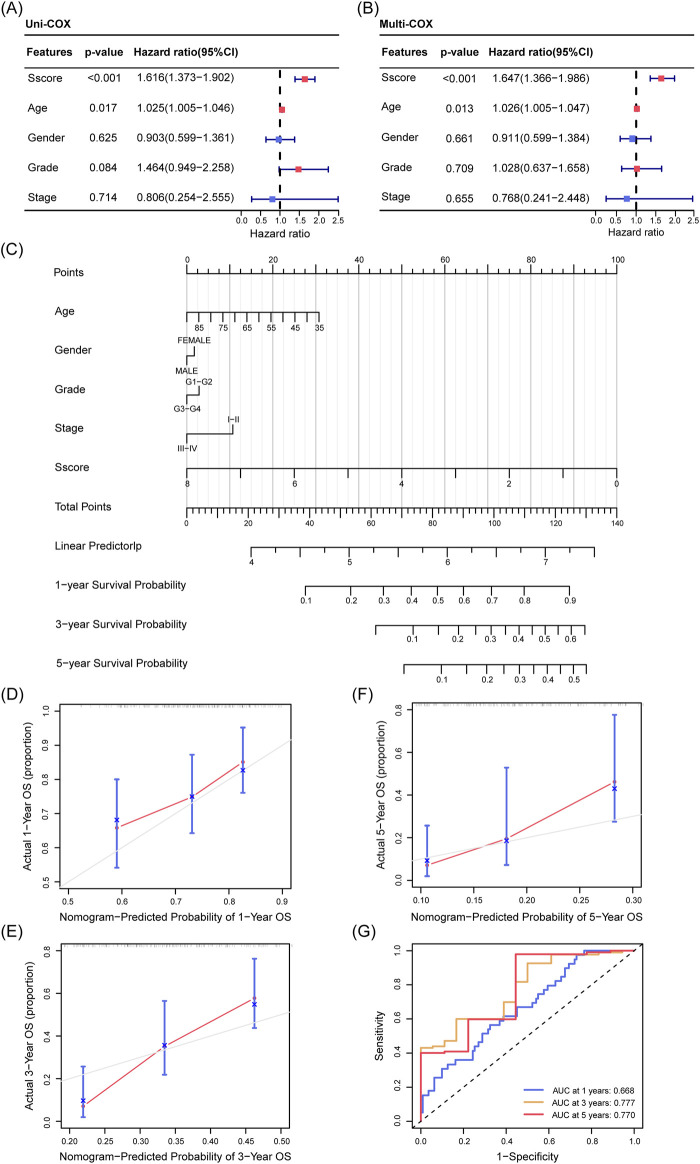
Prognostic Nomogram for OS in PDAC Patients from the TCGA Cohort. **(A, B)** Univariate **(A)** and multivariate **(B)** Cox regression analysis demonstrating the influence of Sscore and clinical characteristics (age, gender, grade, and stage) on OS in PDAC. **(C)** A nomogram incorporating Sscore and clinical factors (age, gender, grade, and stage) to predict 1-, 3-, and 5-year OS in PDAC. **(D–F)** Calibration curves illustrating the agreement between predicted and actual OS for 1 year **(D)**, 3 years **(E)**, and 5 years **(F)**. The gray line represents the ideal prediction, while the blue line shows the observed outcomes, with closer alignment indicating better model performance. **(G)** ROC curves assessing the predictive accuracy of the nomogram for 1-, 3-, and 5-year OS.

To identify significant clinical prognostic factors for PDAC patients, we performed both univariate and multivariate Cox regression analyses. Our results indicated that age (p = 0.017) and Sscore (p < 0.001) were independent predictors of OS (Figures 4A, B). Using the Sscore along with other clinical parameters such as age, gender, tumor grade, and stage, we constructed a nomogram—a predictive tool used to estimate patient outcomes. This nomogram was designed to predict the 1-, 3-, and 5-year OS of PDAC patients (Figure 4C). The calibration curves demonstrated a strong agreement between predicted and actual survival rates (Figures 4D-F). Additionally, ROC curve analysis revealed high predictive accuracy, with areas under the curve (AUC) values of 0.668, 0.777, and 0.770 for 1-, 3-, and 5-year survival, respectively (Figure 4G). Overall, the multivariate Cox regression and nomogram analyses identified key prognostic indicators for PDAC and validated the utility of Sscore, providing a valuable reference for personalized treatment planning and clinical decision-making.

### 3.4 Increased genome variation in high-Sscore patients

**FIGURE 5:**
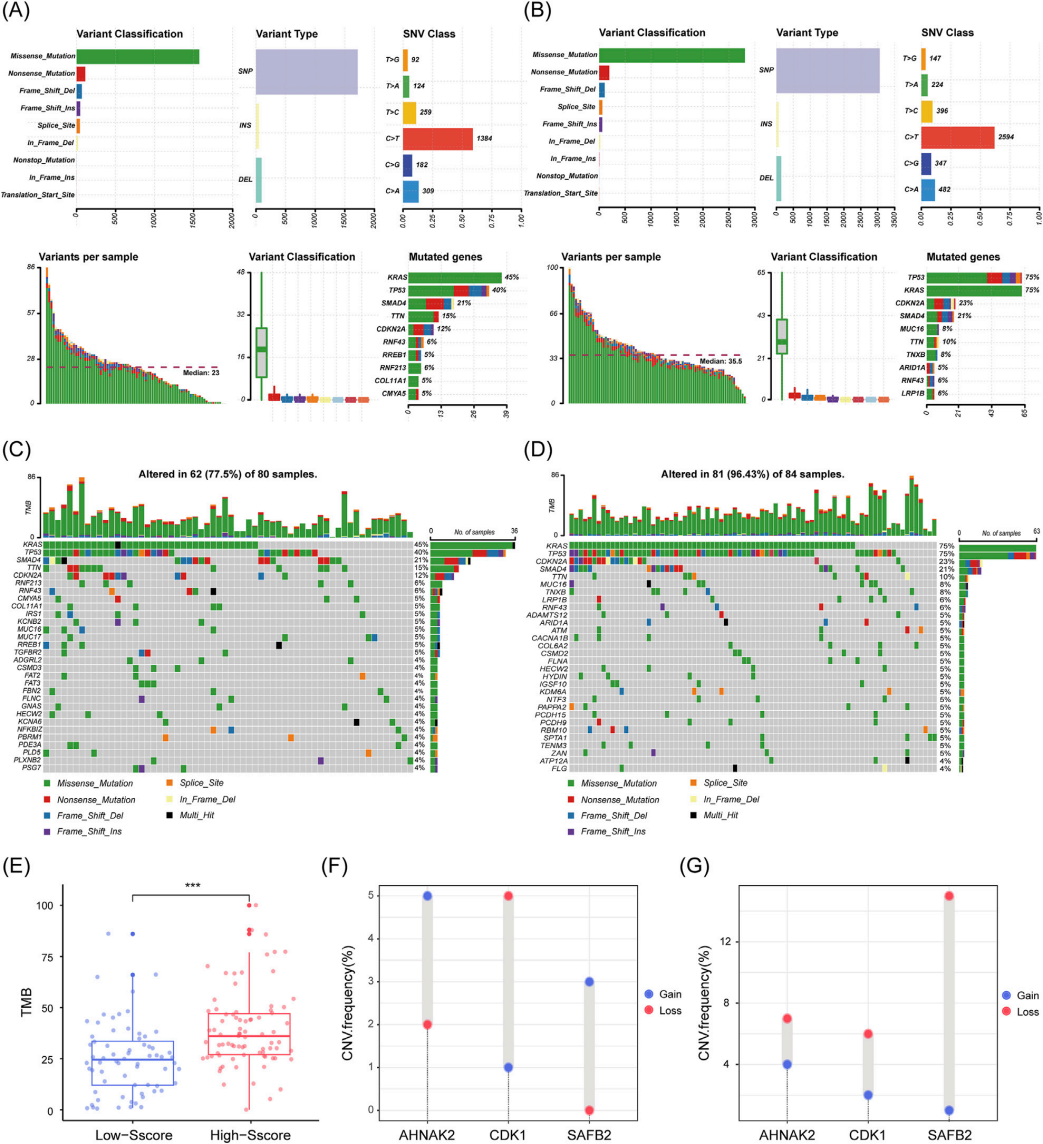
Increased Genome variation in High Sscore Patients. **(A–D)** Genomic mutation characteristics in low and high Sscore patients. **(A)** shows the low Sscore group, and **(C)** shows the high Sscore group. The upper left bar chart illustrates the distribution of SNPs, insertions (INS), and deletions (DEL). The SNV class denotes the relative proportions of base changes, with C>T transitions being the most frequent. The lower left panel depicts the number of variants per sample, while the box plot below shows the distribution of mutation types. **(B, D)** are waterfall plots showing mutations in low Sscore **(B)** and high Sscore **(D)** patients. The top bar charts indicate TMB, while the numbers on the right represent mutation frequencies for each gene. **(E)** Total TMB in high and low Sscore patients. p-values were derived from Mann-Whitney U test. **(F, G)** CNV frequencies of *CDK1*, *AHNAK2*, and *SAFB2* in low Sscore **(F)** and high Sscore **(G)** patients. ***p < 0.001.

After confirming the significance of the Sscore in PDAC, we investigated the potential mechanisms underlying the prognostic differences observed in High and Low-Sscore patients. Since DE-SSEGs are involved in chromatin remodeling and DNA damage repair, we analyzed genome variation between these two groups. Somatic mutation analysis revealed that missense mutations, primarily in the form of single nucleotide polymorphisms (SNPs), were the predominant mutation type in both groups, with similar SNP distribution patterns (Figures 5A, C). However, High-Sscore patients exhibited significantly higher mutation frequencies in well-known PDAC-associated genes, including *KRAS* (75% vs. 45%), *TP53* (40% vs. 23%), *CDKN2A* (23% vs. 21%), and *SMAD4* (21% vs. 12%), compared to Low-Sscore patients (Figures 5B, D). Moreover, High-Sscore patients displayed a markedly higher total number of somatic mutations (35.5 vs. 23) and increased TMB, with significant differences in the mutation rates of key genes (p < 0.001; Figure 5E). Of particular note, CNV analysis of the three key prognostic genes in the Sscore model revealed that the deletion frequency of *SAFB2* was substantially higher in High-Sscore patients, suggesting that loss of *SAFB2* may contribute to tumor progression (Figures 5F, G). In conclusion, our findings demonstrate a strong association between the Sscore and genome variation in PDAC patients, highlighting *SAFB2* as a critical target for further investigation in tumor progression.

### 3.5 Lower immune infiltration and diminished immunotherapy response in high-Sscore patients

**FIGURE 6:**
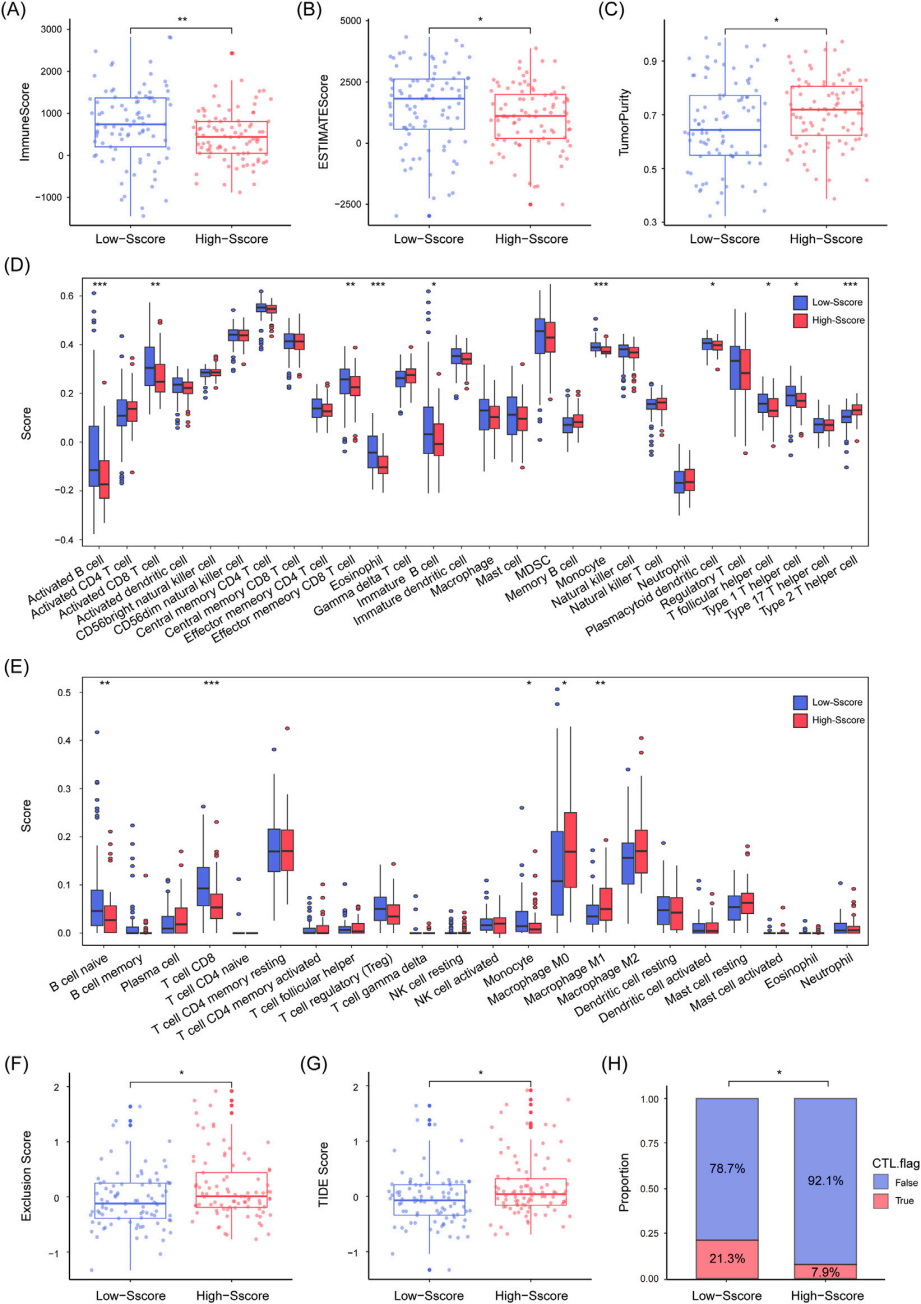
Lower Immune Infiltration and Diminished Immunotherapy Response in High Sscore Patients. **(A–C)** Immune score **(A)** and ESTIMATE score **(B)** are notably higher in low Sscore patients, while tumor purity **(C)** is lower. **(D, E)** ssGSEA **(D)** and CIBERSORT **(E)** algorithms illustrate the distribution and infiltration patterns of different immune cell types in high and low Sscore patients. **(F–H)** TIDE analysis, including exclusion score **(F)**, TIDE score **(G)**, and CTL infiltration status **(H)**. Red indicates presence (True), blue indicates absence (False) in bar graph. Statistical analyses include Mann-Whitney U test **(A–G)**, Chi-square test **(H)**, *p < 0.05, **p < 0.01, ***p < 0.001.

It has been previously established that SUMOylation plays a pivotal role in regulating the tumor microenvironment (TME), immune checkpoints, and immune cell activity, which in turn affects tumor immune evasion (Gu et al., 2023; Xie et al., 2024). TMB is another well-recognized biomarker for predicting the response to immunotherapy (Jardim et al., 2021). Based on these findings, we hypothesized that the Sscore might modulate PDAC prognosis by influencing TME characteristics. To investigate this, we applied three algorithms (“ESTIMATE”, “CIBERSORT”, and “ssGSEA”) to compare the composition and immune profiles of TME in High and Low-Sscore patients. Our results showed that Low-Sscore patients had lower tumor purity but higher immune and ESTIMATE scores, indicating a more complex immune microenvironment (Figures 6A–C). Specifically, Low-Sscore patients displayed higher levels of immune regulatory cells, such as plasmacytoid dendritic cells and follicular helper T cells, along with immune effector cells, including TH1 cells, activated CD8^+^ T cells, and activated B cells. In contrast, High-Sscore patients had greater infiltration of macrophages and TH2 cells (Figures 6D, E). Furthermore, the TIDE algorithm revealed that High-Sscore patients had significantly higher TIDE and exclusion scores, suggesting a greater risk of immune escape and markedly reduced infiltration of cytotoxic T lymphocytes (CTLs) (Figures 6F–H). These findings imply that High-Sscore patients exhibit stronger immunosuppressive characteristics and may have a reduced likelihood of benefitting from immunotherapy.

### 3.6 Increased drug resistance in High-Sscore patients

**FIGURE 7:**
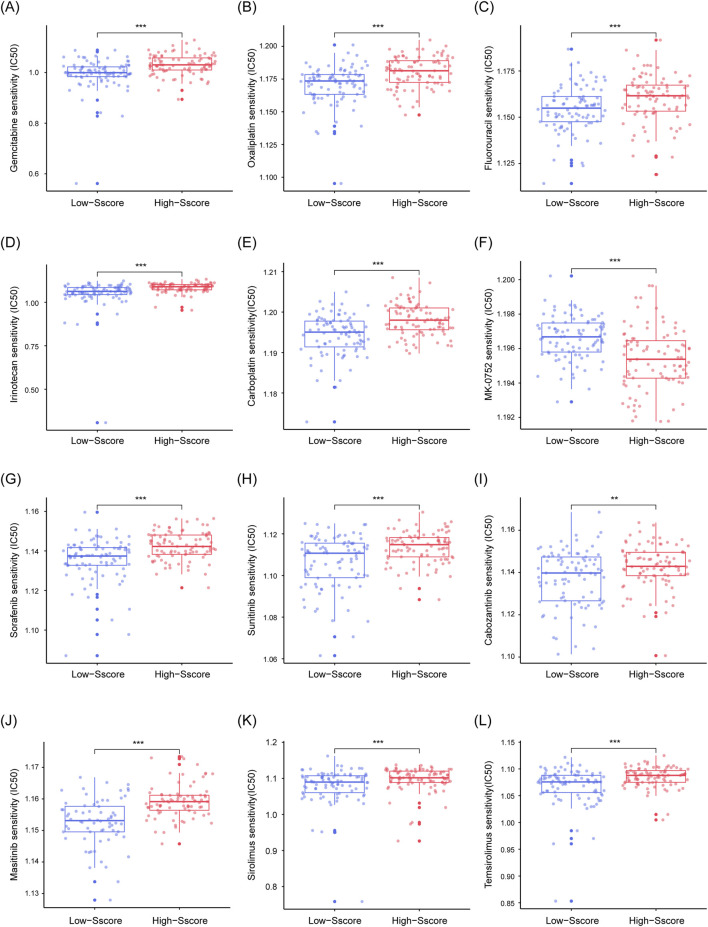
Decreased Drug Sensitivity in High Sscore Patients. **(A–L)** Box plots illustrate the estimated IC50 values of five standard chemotherapy drugs (gemcitabine **(A)**, oxaliplatin **(B)**, fluorouracil **(C)**, irinotecan **(D)**, and carboplatin **(E)**), a NOTCH pathway inhibitor (MK-0752 **(F)**), four tyrosine kinase inhibitors (sorafenib **(G)**, sunitinib **(H)**, cabozantinib **(I)**, and masitinib **(J)**), and two mTOR pathway inhibitors (rapamycin **(K)** and sirolimus **(L)**). Drug sensitivity analysis for targeted therapies was performed using the Cancer Therapeutics Response Portal (CTRP). p-values were derived from Mann-Whitney U test, **p < 0.01, ***p < 0.001.

It is well-established that genome variation and complexity of the TME both play critical roles in the development of drug resistance in tumors. Genome variation can influence not only the biological characteristics of tumor cells but also their response to treatment by modifying the composition and function of TME. In addition, some mutations may directly cause acquired resistance by promoting the expression of resistance-related genes or enhancing the tumor cells’ ability to adapt to therapies. Given this, we explored the relationship between the Sscore and drug sensitivity in PDAC, particularly with conventional chemotherapies and targeted therapies. Based on the 2023 ESMO Pancreatic Cancer Clinical Treatment Guidelines (Conroy et al., 2023), we analyzed the IC50 of five common chemotherapy drugs (gemcitabine, oxaliplatin, fluorouracil, irinotecan, and carboplatin), a NOTCH pathway inhibitor (MK-0752), two mTOR inhibitors (rapamycin and sirolimus), and four tyrosine kinase inhibitors (sorafenib, sunitinib, cabozantinib, and masitinib). Our findings demonstrated that, except for MK-0752, patients with high Sscore displayed significantly higher resistance to all of the drugs tested (Figures 7A–L). This suggests that activation of the NOTCH pathway might be a contributing factor to the increased drug resistance observed in these patients. Therefore, the Sscore is closely linked to drug resistance in PDAC, providing valuable insights for guiding clinical treatment strategies.

### 3.7 scRNA-seq reveals strong correlation between high Sscore and malignant characteristics of PDAC cells

**FIGURE 8:**
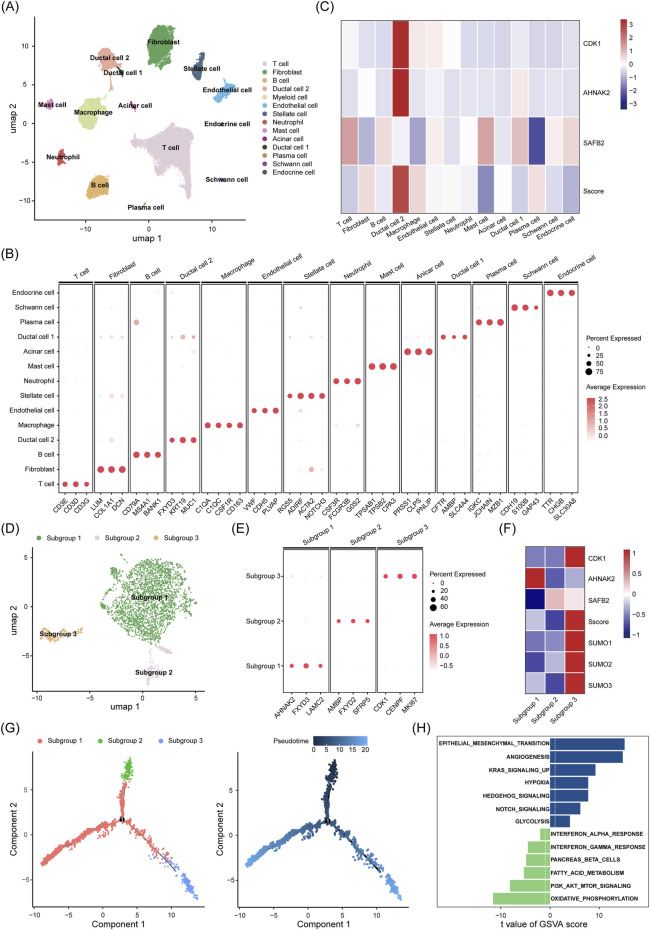
scRNA-seq Reveals the Distribution of Sscore and its Biological Relevance in PDAC. **(A)** UMAP plot illustrates the distribution of 14 major cell types within the dataset (GSE212966). **(B)** A dot plot visualizes the expression levels of characteristic marker genes in each cell type. **(C)** The expression patterns and overall scores of the three key genes in the Sscore model (*CDK1*, *AHNAK2*, *SAFB2*) across various cell types. **(D)** UMAP plot shows the clustering of ductal cells into three distinct subpopulations following dimensionality reduction. **(E)** A dot plot displays the characteristic marker gene expression levels in each of the three cell subpopulations. **(F)** The expression distribution of the three Sscore model genes (*CDK1*, *AHNAK2*, *SAFB2*), SUMOylation substrate encoding genes (*SUMO1*, *SUMO2*, *SUMO3*) and Sscore across the three cell subpopulations. **(G)** Pseudotime trajectory analysis of the three ductal cell subpopulations. **(H)** GSVA analysis reveals the pathway enrichment between high and low Sscore cell subpopulations.

Due to the complex TME of PDAC, we utilized scRNA-seq data from GSE212966, which includes six PDAC and three adjacent normal tissue samples (Supplementary Figure S3A; Chen et al., 2023). After quality control and removal of doublets, 39,786 cells were obtained. Using cell cluster-specific markers, we identified 14 subgroups: ductal cells (type 1 and type 2), T cells, endothelial cells, stellate cells, fibroblasts, B cells, plasma cells, macrophages, neutrophils, mast cells, acinar cells, endocrine cells, and Schwann cells (Figures 8A, B; Supplementary Figure S3B). Notably, we identified two ductal cell types in PDAC. Type 2 ductal cells exhibited elevated expression of malignant markers (*MUC1*, *FXYD3*, *KRT19*), while type 1 ductal cells expressed normal pancreatic markers (*CFTR*, *AMBP*, *SLC4A4*) (Figure 8B). Moreover, type 1 ductal cells and acinar cells were primarily from normal tissues, while type 2 ductal cells were mainly from tumor samples (Supplementary Figure S3A), suggesting type 2 cells as a malignant population. Chromosomal CNV analysis showed significantly higher CNV levels in type 2 than type 1 ductal cells (Supplementary Figure S3C), further supporting their role as the major tumor population in PDAC.

Next, we explored the distribution of three key genes in the Sscore model (*CDK1*, *AHNAK2*, *SAFB2*) across cell types. Heatmap analysis revealed that type 2 ductal cells had the highest Sscore, with elevated *CDK1* and *AHNAK2*, while *SAFB2* was primarily expressed in T cells and mast cells, and significantly lower in type 2 than type 1 ductal cells (Figure 8C). Consistent results were obtained from another single-cell dataset (GSE194247) (Supplementary Figures S4A–D).

Subsequently, we further divided the ductal cells into three subgroups, demonstrating distinct marker expression patterns and Sscore (Figures 8D–F). Subgroup 2 was predominantly composed of type 1 ductal cells, whereas type 2 ductal cells were distributed into subgroups 1 and 3. Notably, subgroup 3 exhibited a higher Sscore, along with elevated expression of SUMOylation modifier factors (*SUMO1/2/3*) and key cell cycle regulators (*MKI67*, *CDK1*, *CENPF*), indicating enhanced proliferative capacity. Given the critical role of SUMOylation in stabilizing cell cycle proteins and promoting mitotic progression, these findings imply that increased SUMOylation activity may contribute to the heightened proliferation observed in this subgroup. Moreover, the elevated expression of *SUMO1/2/3* in malignant ductal cells compared to normal ductal cells further underscores the potential role of SUMOylation in promoting tumor progression (Supplementary Figure S4D). In contrast, subgroup 1 had high oncogene expression (*AHNAK2*, *FXYD3*, *LMC2*), reflecting changes in cytoskeletal regulation and extracellular matrix remodeling. Pseudotime analysis showed ductal cells progressing from subgroup 2 to subgroups 1 and 3, with subgroup 3 likely evolving from subgroup 2 (Figure 8G). Finally, GSVA analysis revealed significant metabolic pathway enrichment differences between high and low Sscore subgroups (glycolysis, oxidative phosphorylation, and fatty acid metabolism). High Sscore subgroup exhibited enhanced angiogenic potential and a greater propensity for epithelial mesenchymal transition (EMT), whereas low Sscore subgroup was enriched in the PI3K/AKT/mTOR signaling pathway and demonstrated stronger interferon α and γ responsiveness (Figure 8H). Together, these findings suggest that subgroup 3 harbors greater malignancy and immunosuppressive properties, aligning with observed immune infiltration patterns and drug sensitivity profiles in High-Sscore patients.

### 3.8 SAFB2 suppresses PDAC cell proliferation, invasion, and migration

**FIGURE 9:**
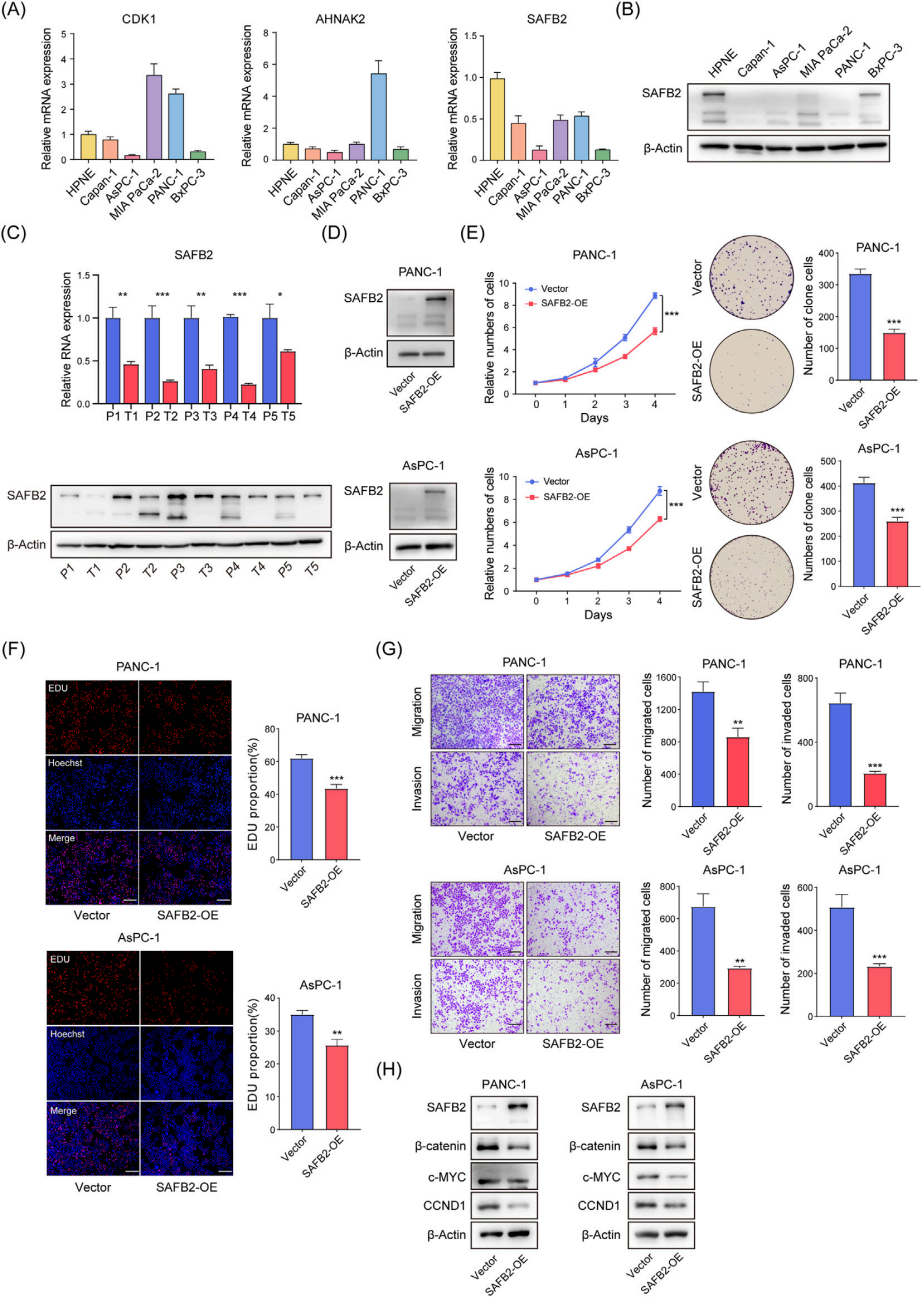
*SAFB2* Inhibits PDAC Cell Proliferation, Invasion, and Migration. **(A)** RT-qPCR analysis of *CDK1*, *AHNAK2*, and *SAFB2* expression levels in PDAC cell lines and normal pancreatic ductal epithelial cells. **(B)** Western blot analysis of SAFB2 protein levels in PDAC cell lines and normal pancreatic ductal epithelial cells. **(C)** The expression of SAFB2 in five pairs of PDAC tissues and their corresponding adjacent normal tissues **(D)** Western blot validation of successful construction of SAFB2 overexpression models in the PANC-1 and AsPC-1 cell line. **(E)** Proliferation curves and colony formation assay of *SAFB2*-overexpressing PANC-1 and AsPC-1 cells, with empty vector-transfected PANC-1 and AsPC-1 cells as controls. **(F)** EdU assay assessing the proliferation of *SAFB2* overexpression clones, with empty vector-transfected PANC-1 and AsPC-1 cells as controls. Scar bar: 30 μm. **(G)** Transwell assay showing the invasion and migration abilities of *SAFB2* overexpression clones, with empty vector-transfected PANC-1 and AsPC-1 cells as controls. Scar bar: 30 μm. **(H)** Western blot analyzed the protein levels of Wnt/β-catenin signaling-associated factors of *SAFB2* overexpression clones, with empty vector-transfected PANC-1 and AsPC-1 cells as controls. Scar bar: 30 μm. Statistical analyses were derived from Student’s t-tests, error bars represent SD based on three independent experiments. ^ns^p > 0.05, *p < 0.05, **p < 0.01, ***p < 0.001.

To investigate the impact of key Sscore genes on PDAC, we evaluated the expression of *CDK1*, *AHNAK2*, and *SAFB2* in PDAC cells. RT-qPCR and Western blot results demonstrated that SAFB2 expression was notably reduced in PDAC cell lines compared to normal pancreatic ductal epithelial cells (Figures 9A, B). Consistently, comparative analyses of five paired adjacent normal and PDAC tissues corroborated these findings (Figure 9C). Among the genes comprising the Sscore, *SAFB2* stands out as the feature gene with the highest weight, exhibiting significant upregulation in PDAC cells and tissues. Furthermore, given its significant downregulation and the clear CNV deletions observed in High-Sscore patients, we prioritized *SAFB2* as a key target for further investigation. To assess its functional role, we constructed *SAFB2* overexpression models in PANC-1 and AsPC-1 cell lines (Figure 9D). Functional assays, including CCK8, colony formation assays and EdU demonstrated that *SAFB2* overexpression significantly inhibited PDAC cell proliferation (Figures 9E, F). Transwell assays further confirmed that *SAFB2* overexpression significantly reduced the migration and invasion abilities of PDAC cells (Figure 9G). These findings suggest that *SAFB2* may play a critical role in suppressing the malignant properties of PDAC cells.

Given that *SAFB2* has been reported to inhibit breast cancer progression through the Wnt/β-Catenin pathway, we further explored its role in PDAC. Western blot analysis revealed that *SAFB2* overexpression significantly reduced β-Catenin expression and transcriptional activity (Figure 9H), indicating that *SAFB2* suppresses PDAC progression, at least in part, by inhibiting the Wnt/β-Catenin pathway.

## 4 Discussion

PDAC remains one of the most challenging cancers to treat due to its poor prognosis, difficulty in early detection, low surgical resection rates, poor responsiveness to treatment, and high recurrence and metastasis rates (Conroy et al., 2023; Peng et al., 2019). Early diagnosis and targeted therapies are thus critical but unmet needs in PDAC management. Our study identified significantly elevated levels of SUMOylation in PDAC cells, with SUMOylation inhibition effectively reducing PDAC cell proliferation. The Sscore model developed based on SSEGs not only demonstrated robust predictive capabilities but also provided insights into PDAC patients’ genome variation, immune microenvironment composition, and drug sensitivities. Moreover, the downregulation of *SAFB2* in PDAC cells suggests that it may exert tumor-suppressive effects by inhibiting the Wnt/β-Catenin pathway, pointing to potential novel therapeutic avenues for PDAC.

### 4.1 Advances in prognostic prediction of PDAC using SSEGs

IHC analysis and *in vitro* experiments showed a marked increase in SUMOylation levels in PDAC tissues. The SUMOylation inhibitor TAK-981 suppresses PDAC progression by disrupting cell cycle dynamics. Specifically, TAK-981 selectively targets the SUMO pathway by forming a covalent complex with SUMO, effectively inhibiting SUMO E1 activity while preserving ubiquitin E1 function (Langston et al., 2021). Treatment with TAK-981 led to a dose-dependent reduction in PDAC cell proliferation (Kumar et al., 2022). Furthermore, PDAC cell lines exposed to TAK-981 displayed an accumulation of cells in the G2/M phase, indicating mitotic arrest and impaired cell cycle progression. This confirms the validity of predicting PDAC prognosis using SSEGs. Several studies have highlighted the pivotal role of SUMOylation in PDAC progression. For instance, SENP3 suppresses PDAC metastasis by deSUMOylating DKC1, while SUMOylation of XRCC4 at K115 enhances nuclear localization on chromatin, contributing to oxaliplatin resistance. Furthermore, inhibiting SUMOylation in PDAC activates interferon pathways, boosting antitumor immunity (Kumar et al., 2022; Wu et al., 2023; Zheng et al., 2023). Based on 134 DE-SSEGs, we developed a prognostic model, the Sscore, which demonstrated superior predictive performance compared to models using DNA methylation driver genes and anoikis-related genes. Overall, the Sscore effectively assesses PDAC prognosis and highlights the potential of targeting SUMOylation for therapeutic interventions.

### 4.2 Mechanisms underlying SUMOylation-mediated PDAC risk

Given the importance of SUMOylation in DNA repair and cell cycle regulation, we hypothesize that dysregulation of SSEGs in high Sscore patients could lead to the accumulation of somatic mutations, activating oncogenes and inactivating tumor suppressor genes, thereby promoting tumor progression. Genome variation are also linked to immune evasion, drug resistance, and prognosis. Our study found that high Sscore patients exhibited higher frequencies of somatic mutations, with significantly elevated TMB and mutation rates in key genes compared to low Sscore patients. Notably, mutations in KRAS, TP53, CDKN2A, and SMAD4 were more prevalent in high Sscore patients, suggesting a more aggressive tumor phenotype. CNV analysis further revealed a higher frequency of *SAFB2* deletions in high Sscore patients, implicating *SAFB2* as a potential tumor suppressor in PDAC. In conclusion, aberrant SUMOylation likely exacerbates genomic instability in PDAC, and the Sscore model effectively stratifies patients based on their levels of genome variation.

It is now well-established that tumor progression is the result of continuous interactions between tumor cells and the surrounding TME. Immune cells, as a crucial component of the TME, can either facilitate or inhibit tumor growth (Mao et al., 2021; Lei et al., 2020; Hinshaw and Shevde, 2019). Studies have demonstrated that SUMOylation directly regulates immune cells (such as dendritic cells, CD8^+^ T cells, Treg cells, and macrophages), impacting their immune functions (Sun et al., 2023; Yu et al., 2018; Xiao et al., 2022; Hu et al., 2021; Wu et al., 2022; Lightcap et al., 2021). Our research revealed that PDAC patients with lower Sscore exhibited reduced tumor purity, higher immune and ESTIMATE scores, and greater infiltration of immune-related cells in the TME compared to high Sscore patients. Similarly, TIDE analysis suggested that high Sscore patients had a diminished response to immunotherapy, with a lower likelihood of benefiting from such treatment. Paradoxically, high Sscore patients showed increased TMB levels, a marker typically associated with improved immunotherapy outcomes (Gu et al., 2022). This contradiction could be explained by the immunosuppressive TME in PDAC, the presence of immune evasion mechanisms in tumor cells, or the insufficient immunogenicity of certain tumor mutations (Jiang et al., 2016; Wang-Gillam et al., 2022; Cao et al., 2021; Piersma et al., 2020). Thus, despite elevated TMB, high Sscore patients may not respond favorably to immunotherapy.

Additionally, SUMOylation has been implicated in directly mediating drug resistance in tumor cells (Gu et al., 2024). Genome variation and TME modifications, both closely linked to SUMOylation, also contribute to acquired resistance in tumors (Gu et al., 2023; Wu et al., 2022; Xia et al., 2022). Our analysis of drug sensitivity demonstrated that the Sscore reliably predicts the response of PDAC patients to various treatments. High Sscore patients exhibited significant resistance to commonly used chemotherapeutic agents, mTOR inhibitors, and tyrosine kinase inhibitors but showed increased sensitivity to NOTCH pathway inhibitors, suggesting potential NOTCH pathway activation. This aligns with previous findings, where SUMOylation of N1ICD was shown to enhance downstream NOTCH signaling (Zhu et al., 2017).

The TME of PDAC is highly complex, and traditional transcriptomic methods struggle to fully capture the intercellular diversity. Through single-cell transcriptomics, we identified that malignant ductal cells exhibited significantly higher Sscore values than normal ductal cells. Specifically, type 2 ductal cells with a high Sscore exhibited elevated expression of SUMOylation modifier factors (SUMO1/2/3) alongside increased expression of cell cycle-related genes, highlighting the crucial role of SUMOylation in cell cycle progression and proliferation. Subcluster analysis combined with GSVA revealed that these cells were at the terminal end of the ductal cell differentiation trajectory and possessed more pronounced malignant characteristics. High Sscore subgroups were significantly enriched in pro-tumor pathways such as angiogenesis, epithelial-mesenchymal transition (EMT), and the NOTCH and Hedgehog pathways. Conversely, low Sscore subgroups showed enrichment in the p53 and PI3K/AKT/mTOR signaling pathways and exhibited strong interleukin α and γ responses, retaining partial pancreatic normal cell functions. scRNA-seq highlighted a strong link between the Sscore and PDAC cell malignancy, suggesting that high Sscore subgroups harbor more aggressive tumor biology and immunosuppressive environments, aligning with previous findings of immune evasion and drug resistance in high Sscore patients.

Previous studies have demonstrated that dysregulation of the SUMOylation pathway induces drug resistance and immune evasion in tumor cells (Seeler and Dejean, 2017). By modulating the stability and activity of proteins involved in key signaling pathways such as NOTCH, p53, NF-κB, and Wnt/β-Catenin, SUMOylation contributes to chemoresistance and immune escape, making it a promising therapeutic target (Seeler and Dejean, 2017; Zhu et al., 2017; Boulanger et al., 2019; Kukkula et al., 2021). Therefore, integrating SUMOylation inhibition with existing treatment strategies may enhance antitumor efficacy and help overcome resistance mechanisms. Currently, TAK-981 is the only SUMOylation inhibitor that has advanced to phase I/II clinical trials, with studies targeting both hematologic malignancies and advanced or metastatic solid tumors (Boulanger et al., 2019). Ongoing trials are evaluating its efficacy in combination with immune checkpoint inhibitors (NCT04381650) and monoclonal antibodies targeting CD38 and CD20 (NCT04776018, NCT04074330) for hematologic cancers. Additionally, TAK-981 is being investigated in combination with immunotherapy (avelumab) and anti-EGFR antibodies for solid tumors (NCT04065555), with a particular focus on its role in modulating the tumor microenvironment. These trials provide preliminary clinical validation of SUMOylation inhibition as a therapeutic approach; however, further research is required to elucidate its precise mechanisms and identify potential synergistic treatment strategies.

### 4.3 The role of key genes in sscore and their association with PDAC

The Sscore model is based on three critical genes: *CDK1*, *AHNAK2*, and *SAFB2*. Cyclin-dependent kinases (CDKs) are crucial proteins that, when forming complexes with cyclins, drive the progression of the cell cycle (Santamaría et al., 2007). CDK1, a central member of this kinase family, plays a pivotal role in cell cycle regulation, checkpoint activation, and DNA damage repair (Wang et al., 2023). Numerous studies have demonstrated the upregulation of *CDK1* in various cancers, where its dysregulation is closely linked to tumor development and progression (Wang et al., 2023). Currently, *CDK1* inhibitors, such as Rigosertib and Zotiraciclib, are in Phase III clinical trials and have shown potential in treating PDAC and gliomas (O'Neil et al., 2015; Le Rhun et al., 2024). AHNAK, part of the large nucleoprotein family, is involved in multiple physiological and pathological activities, including lipid metabolism, membrane repair, and tumor migration (Davis et al., 2014). Recent research has underscored *AHNAK2* as a prognostic marker for cancers such as thyroid cancer, melanoma, PDAC, and bladder cancer (Zhang et al., 2023). In PDAC, elevated *AHNAK2* levels promote tumor progression by preventing c-MET degradation, thereby sustaining HGF/c-MET pathway activation (Chen et al., 2024). Knockdown of *AHNAK2* has been shown to impede tumor progression through inhibition of the NF-κB/MMP-9 signaling pathway (Tang et al., 2024).

*SAFB2*, identified as a key SSEG in this study, plays a critical role in PDAC development. As mentioned earlier, *SAFB2* is part of the *SAFB* family of nuclear proteins, which participate in essential biological processes, including RNA processing, cell proliferation, stress responses, and apoptosis. *SAFB2* acts as a transcriptional repressor by binding to chromatin and interacting with regulatory proteins such as transcription factors and chromatin remodeling complexes. SUMOylation enhances the transcriptional repression ability of *SAFB2*, particularly immune-related genes like *MHC-I*, enabling tumor cells to evade immune detection by reducing antigen presentation, thus promoting immune escape (Garee et al., 2011; Demel et al., 2022). Interestingly, research by Liu et al. shows that SUMOylated *SAFB* can promote ribosomal gene transcription by recruiting RNA polymerase II, which aids in pre-mRNA splicing (Liu et al., 2015). This divergence in function may be due to different cellular environments or external conditions that influence how SUMOylated *SAFB* operates. Despite the high sequence similarity between *SAFB1* and *SAFB2*, they differ notably in their gene regulatory roles. Unlike SAFB1, which is strictly nuclear, SAFB2 can also localize in the cytoplasm and interact with proteins like Vinexin-β, suggesting a role in signaling pathways and cytoskeletal organization (Hutter et al., 2020). Additionally, beyond repressing AR and ERα activity, *SAFB2* has been shown to suppress breast cancer progression by inhibiting the Wnt/β-catenin pathway via NFAT5 (Zhen et al., 2023).

Nevertheless, the role of SAFB2 in PDAC remains inadequately characterized. Our study revealed significant downregulation of *SAFB2* in PDAC cells. Overexpression of *SAFB2* inhibited cell proliferation, invasion, and migration, and was associated with reduced Wnt/β-Catenin signaling activity. The Wnt/β-Catenin signaling pathway plays a pivotal role in PDAC progression by driving cell cycle regulation and epithelial-mesenchymal transition (Aguilera and Dawson, 2021; Cui et al., 2012; Yang P. et al., 2022). Its aberrant activation not only facilitates tumor initiation but also contributes to therapy resistance, underscoring its significance in PDAC pathophysiology (Cui et al., 2012). These findings suggest a potential link between SAFB2 and Wnt/β-Catenin signaling in tumor suppression, though the underlying mechanism warrants further investigation.

Therefore, future therapeutic strategies could focus on restoring SAFB2 function or targeting its associated pathways to enhance treatment efficacy. Combining Wnt pathway inhibitors (e.g., PRI-724, LGK974) with *SAFB2* overexpression strategies may provide synergistic tumor suppression (Yang P. et al., 2022). Additionally, gene therapy approaches, such as viral vector-mediated SAFB2 delivery or CRISPR activation, could restore SAFB2 function, while epigenetic modulators (e.g., DNA methyltransferase or HDAC inhibitors) may help reactivate its expression. Furthermore, SAFB2 modulation could enhance chemotherapy sensitivity by counteracting Wnt/β-Catenin-driven resistance, potentially improving the efficacy of standard PDAC treatments such as gemcitabine, FOLFIRINOX, or nab-paclitaxel. Future studies should validate these strategies to determine their clinical relevance and therapeutic potential in overcoming PDAC progression and treatment resistance.

### 4.4 Study limitations and future directions

This study employed a retrospective design. Although we validated the Sscore model across several datasets, future prospective studies are essential to confirm its clinical utility. While our findings suggest that *SAFB2* has tumor-suppressive potential in PDAC, its precise role and underlying regulatory mechanisms, particularly in relation to the inhibition of Wnt/β-Catenin signaling and suppression of tumor progression, remain to be fully elucidated. Further research is required to explore the molecular functions of *SAFB2*, particularly regarding its involvement in PDAC progression and its potential as a therapeutic target. In summary, our study presents a novel SUMOylation substrate encoding gene model for predicting PDAC prognosis and unveils *SAFB2* as a promising tumor suppressor. Future investigations should further explore the impact of SUMOylation or SAFB2 modulation in PDAC, paving the way for personalized and targeted therapy innovations.

## 5 Conclusion

**FIGURE 10:**
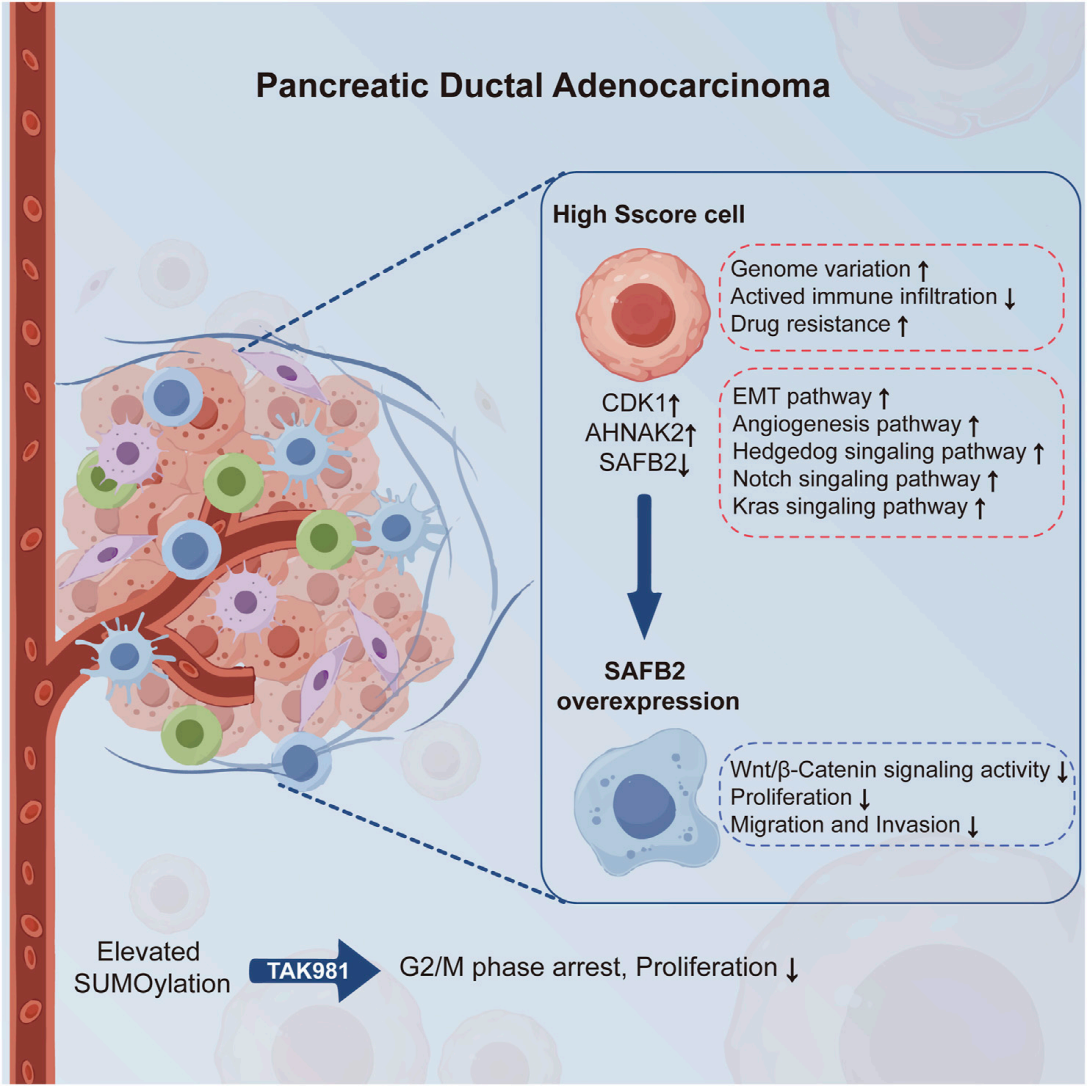
SUMOylation and *SAFB2* in PDAC Progression. This schematic illustrates the role of SUMOylation in promoting PDAC progression and the therapeutic potential of its inhibition via TAK-981, which significantly reduces tumor cell proliferation. High Sscore cells exhibit genome variation, reduced immune infiltration, drug resistance, and activation of pathways such as EMT and angiogenesis. *SAFB2*, downregulated in PDAC, inhibits the progression of PDAC by suppressing the Wnt/β-Catenin signaling pathway, highlighting its tumor-suppressive function.

This study underscores the pivotal role of SUMOylation in the progression of PDAC (Figure 10). We developed the Sscore prognostic model based on essential SUMOylation substrate encoding genes, showing robust predictive accuracy for survival and associations with genome variation, immune infiltration, and drug sensitivity. Single-cell analysis further linked high Sscore to increased tumor malignancy. The observed downregulation of *SAFB2* highlights its potential tumor-suppressive function, with *in vitro* experiments demonstrating its inhibitory effects on PDAC cell proliferation, migration and invasion, possibly through suppression of the Wnt/β-Catenin pathway. Taken together, these findings offer promising directions for advancing personalized and targeted therapies for PDAC.

## Data Availability

The original contributions presented in the study are included in the article/Supplementary Material, further inquiries can be directed to the corresponding author.

## Supplementary material

SUPPLEMENTARY FIGURE S1 Heatmap, PPI Network Analysis, and Prognostic Model Optimization for DE-SSEGs. **(A)** Heatmap depicting DE-SSEG expression in normal pancreatic tissues (GTEx, blue) and PDAC tissues (TCGA-PAAD, red). **(B)** PPI network of the top 30 DE-SSEGs. **(C)** LASSO regression showing the optimal λ value selected via tenfold cross-validation. **(D)** LASSO-Cox analysis results for the eight strongest prognostic DE-SSEGs.

SUPPLEMENTARY FIGURE S2 Expression Patterns of SUMOylation Substrate Encoding Genes and Prognostic Efficiency of the Sscore in LIHC and COAD. **(A, B)** Representative IHC images display SUMO1, SUMO2, and SUMO3 expression in normal liver **(A)** and colon **(B)** tissues, as well as in LIHC **(A)** and COAD **(B)** tissues from the HPA dataset. The violin plots represent relative quantification of SUMO protein expression in these tissues, with p-values derived from Student’s t-tests. **(C, D)** K-M analysis illustrating OS curves and ROC curves assessing the OS prediction for high- and low-Sscore patients in the TCGA-LIHC **(C)** and in the TCGA-COAD **(D)** dataset. **(E, F)** Scatter plots showing risk scores (top), survival time and status (middle), and heatmap of CDK1, AHNAK2 and SAFB2 expression (bottom) for LIHC and COAD patients in the TCGA-LIHC dataset (E), TCGA-COAD dataset **(F)**. ^ns^p > 0.05, **p < 0.01, ***p < 0.001.

SUPPLEMENTARY FIGURE S3 Dimensionality Reduction Clustering and CNV Analysis across the Cells in GSE212966. **(A, B)** UMAP plots demonstrate the distribution of cells from different sample origins **(A)** and the clustering of all cells through dimensionality reduction **(B)**. **(C)** The CNV differences between the two types of ductal epithelial cells were inferred by comparing them to other cell types as a reference.

SUPPLEMENTARY FIGURE S4 Dimensionality Reduction Clustering and Expression Patterns of SUMOylation Substrate Encoding Genes across the Cells in GSE194247. **(A)** UMAP plot illustrates the distribution of major cell types within the dataset (GSE194247). **(B)** UMAP plots demonstrate the clustering of all cells through dimensionality reduction. **(C)** A dot plot visualizes the expression levels of characteristic marker genes in each cell type. **(D)** The expression distribution of the three Sscore model genes (*CDK1*, *AHNAK2*, *SAFB2*), SUMOylation substrate encoding genes (*SUMO1*/*2*/*3*) and Sscore across various cell types.

## Glossary

PDAC: pancreatic ductal adenocarcinoma
NALIRIFOX: nanoliposomal irinotecan, leucovorin, fluorouracil, and oxaliplatin
SUMOylation: small ubiquitin-related modifier
SAFB2: scaffold attachment factor-B2
SLTM: SAF-like transcription modulator
AR: androgen receptors
ERα: estrogen receptors α
TCGA: The Cancer Genome Atlas
GTEx: Genotype-Tissue Expression Project
SSEGs: SUMOylation substrate encoding genes
DEGs: differentially expressed genes
FC: fold change
DE-SSEGs: differentially expressed SUMOylation substrate encoding genes
STRING: Search Tool for Retrieval of Interacting Genes database
PPI: protein-protein interaction
GO: Gene Ontology
KEGG: Kyoto Encyclopedia of Genes and Genomes
HPA: Human Protein Atlas
IHC: immunohistochemistry
OS: overall survival
LASSO: least absolute shrinkage and selection operator
ROC: receiver operating characteristic
ESTIMATE: Estimation of Stromal and Immune cells in Malignant tumors using Expression data
CIBERSORT: cell-type identification by estimating relative subsets of RNA transcripts
ssGSEA: single sample gene set enrichment analysis
GSVA: Gene Set Variation Analysis
TIDE: tumor immune dysfunction and exclusion
TMB: tumor mutational burden
CNV: copy number variation
IC50: half-maximal inhibitory concentration
CTRP: Cancer Therapeutics Response Portal
PCA: principal component analysis
UMAP: uniform manifold approximation and projection
MSigDB: Molecular Signatures Database
ATCC: American Type Culture Collection
SDS-PAGE: SDS-polyacrylamide gel electrophoresis
PVDF: polyvinylidene fluoride
HRP: horseradish peroxidase
K-M: Kaplan-Meier analysis
AUC: areas under the curve
SNPs: single nucleotide polymorphisms
TME: tumor microenvironment
CTLs: cytotoxic T lymphocytes
EMT: epithelial-mesenchymal transition
CDKs: cyclin-dependent kinases
LIHC: Liver hepatocellular carcinoma
COAD: Colon adenocarcinoma
