# Supplementary figures and images for: SUMOylation substrate encoding genes as prognostic biomarkers in pancreatic ductal adenocarcinoma with functional assessment of SAF-B2
Source: Front Pharmacol. 2025 Apr 16;16:1532658. doi: 10.3389/fphar.2025.1532658 (PMC12040899; doi:10.3389/fphar.2025.1532658)

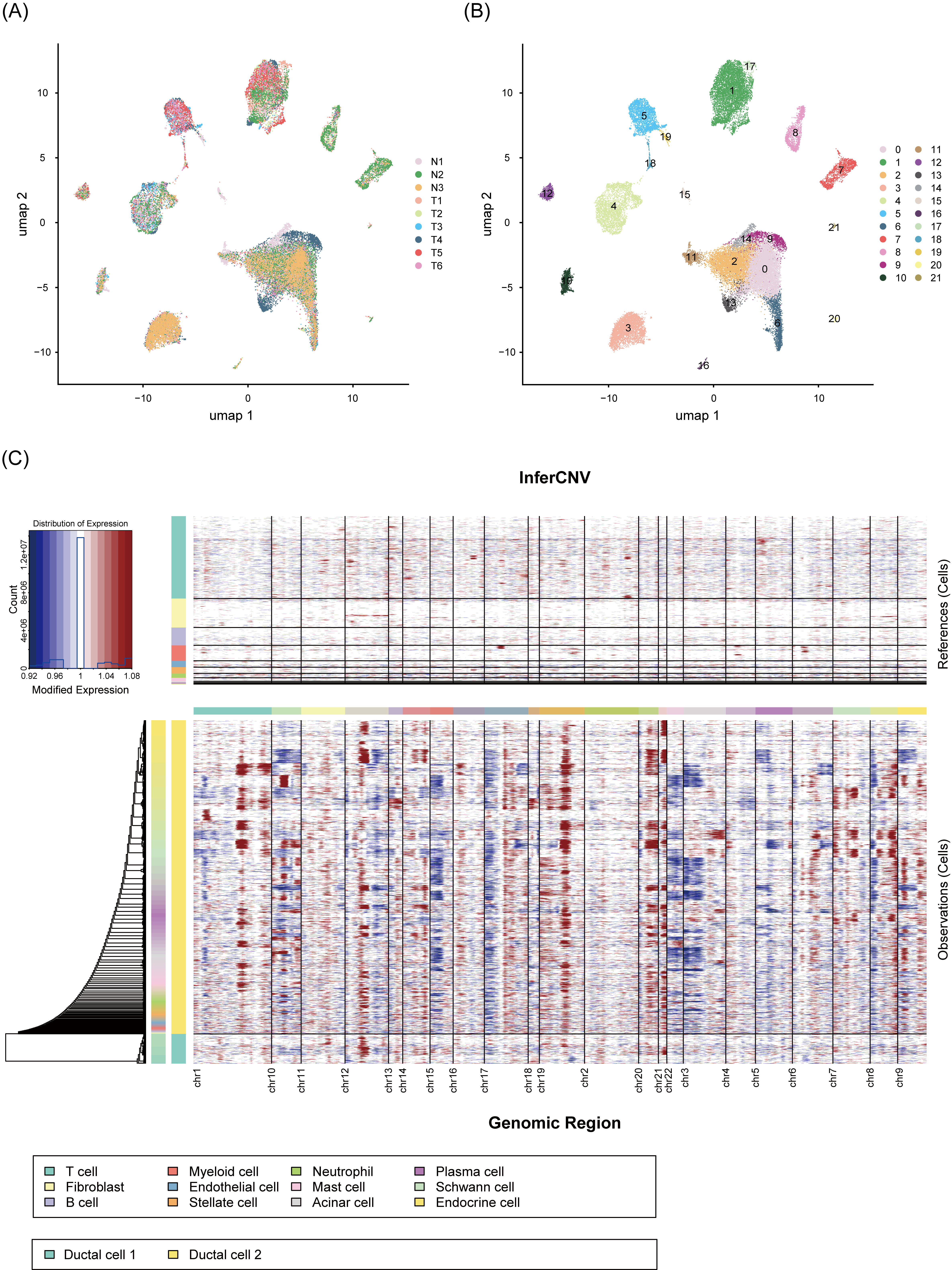

Supplement: Supplementary file 1 [file Image3.tif]

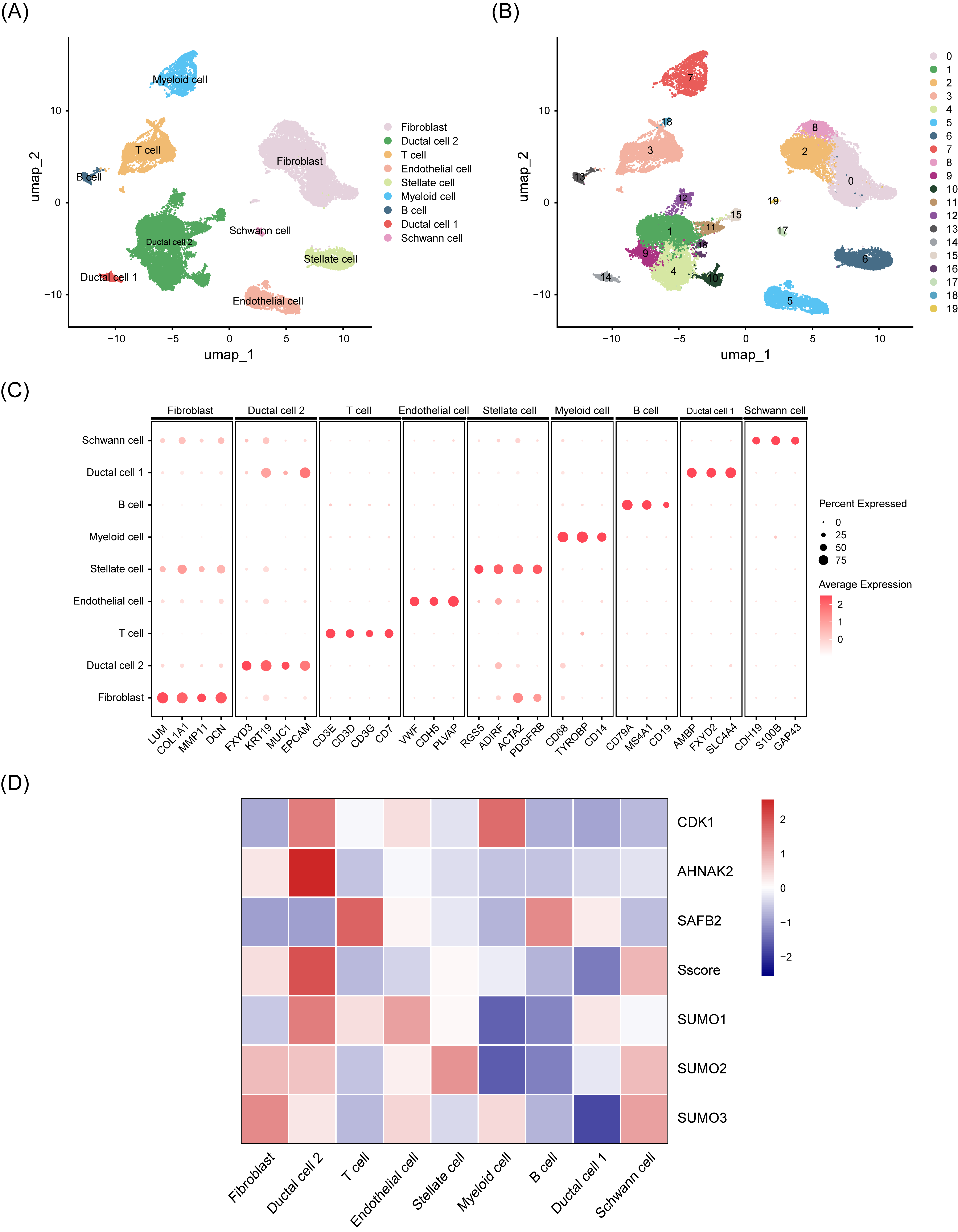

Supplement: Supplementary file 2 [file Image4.tif]

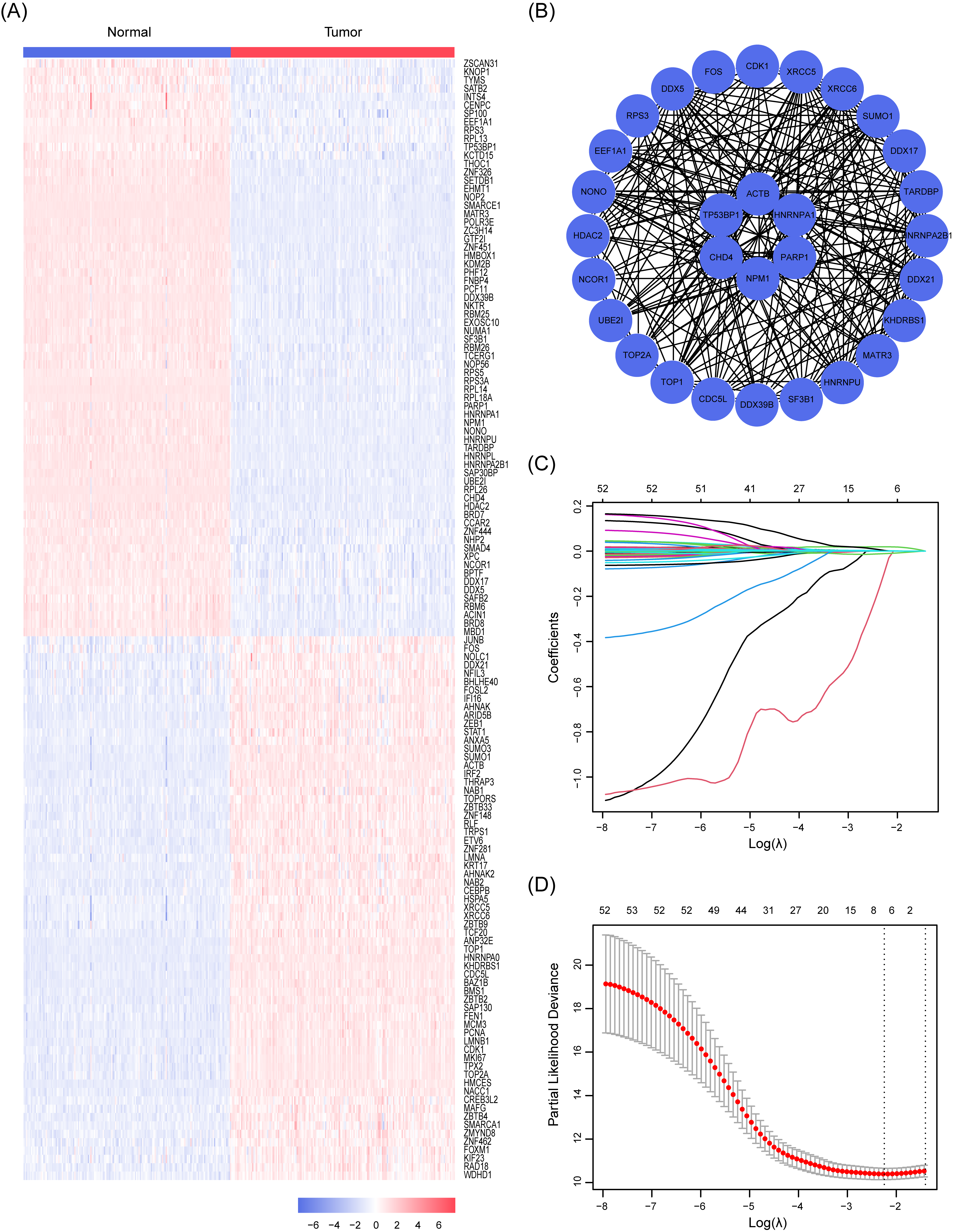

Supplement: Supplementary file 3 [file Image1.tif]

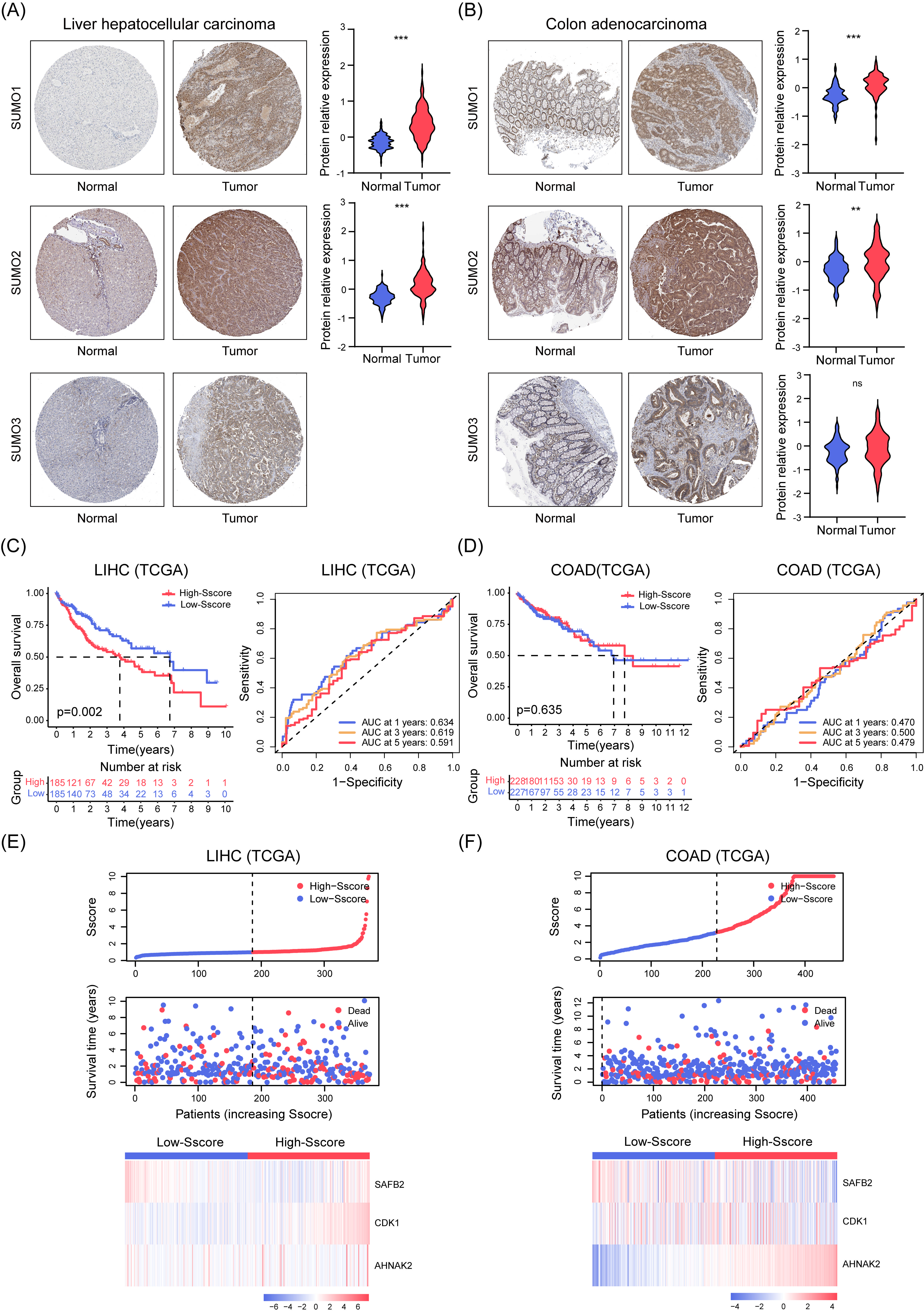

Supplement: Supplementary file 4 [file Image2.tiff]
